# Neural interactions in working memory explain decreased recall precision and similarity-based feature repulsion

**DOI:** 10.1038/s41598-022-22328-4

**Published:** 2022-10-22

**Authors:** Jeffrey S. Johnson, Amanda E. van Lamsweerde, Evelina Dineva, John P. Spencer

**Affiliations:** 1 Department of Psychology, North Dakota State University, Dept. 2765, P.O. Box 6050, Fargo, ND 58108 USA; 2 Center for Visual and Cognitive Neuroscience, North Dakota State University, Fargo, USA; 3 School of Psychology, University of East Anglia, Norwich, UK; 4 Psychological and Brain Sciences, Indiana University, Bloomington, USA

**Keywords:** Human behaviour, Cognitive neuroscience, Computational neuroscience

## Abstract

Over the last several years, the study of working memory (WM) for simple visual features (e.g., colors, orientations) has been dominated by perspectives that assume items in WM are stored independently of one another. Evidence has revealed, however, systematic biases in WM recall which suggest that items in WM interact during active maintenance. In the present study, we report two experiments that replicate a repulsion bias between metrically similar colors during active storage in WM. We also observed that metrically similar colors were stored with lower resolution than a unique color held actively in mind at the same time. To account for these effects, we report quantitative simulations of two novel neurodynamical models of WM. In both models, the unique behavioral signatures reported here emerge directly from laterally-inhibitory neural interactions that serve to maintain multiple, distinct neural representations throughout the WM delay period. Simulation results show that the full pattern of empirical findings was only obtained with a model that included an elaborated spatial pathway with sequential encoding of memory display items. We discuss implications of our findings for theories of visual working memory more generally.

Research has suggested that the ability to actively hold information in visual working memory (VWM) may be limited to as few as 3–4 items^1,2^. For example, studies of change detection^2,3^, which require observers to remember a variable number of simple objects over a brief, unfilled delay, have shown that performance remains quite good for displays containing a small number of items (~ 1–3), but declines rapidly as more to-be-remembered items are added to the memory display (> 4 or so). According to one prominent view, such capacity limits reflect the functioning of a memory system that stores a limited number of fixed-resolution representations in a small number of memory ‘slots’^1–4^. When the number of to-be-remembered items is less than or equal to the number of available slots, each item is stored at a fixed level of high precision in one of the available slots and performance remains good. When the number of items exceeds the number of available slots, however, only a subset of the memory display items is stored and the rest are simply forgotten, negatively impacting performance. Critically, according to the model, information in each slot is stored independently of the other items in the memory display.

Contrary to this view, several lines of research have suggested that memory for individual items is influenced by the properties of the other items being stored. For example, when holding more than one item in memory, individual items are often remembered as being more similar than they really were—that is, individual item representations are biased toward, or ‘attracted’ to, each other^5–8^. This effect has been proposed to reflect the combination of individual item information (e.g., the particular color or orientation of a stimulus) with ensemble statistics reflecting the perceptual average of all of the items in the memory display (e.g., the mean color or orientation of all items) during recall^7^.

More recently, other studies have revealed the opposite pattern: a repulsion bias during multi-item storage in which metrically similar stimuli (e.g., similar colors or orientations) are remembered as being more distinct than they really were^9,10^ (see^11^ for a review of studies showing attraction versus repulsion in WM tasks). Unlike the attraction bias described above, in which individual item representations are thought to be combined with ensemble information during recall, these repulsion biases have been proposed to arise as a result of direct interactions among memory items during active storage. To test this possibility, Scotti and colleagues^10^ used a non-retinotopic VWM paradigm (that is, item location was varied from study to test) and manipulated the duration of the memory delay and the necessity of actively attending to the items throughout the delay. Results showed the predicted repulsion bias for similar items with longer delays when the items were actively attended. A much smaller repulsion effect in a short-delay condition suggested that, rather than reflecting a perceptual bias, the observed repulsion bias likely arose as a result of metric-dependent interactions occurring among items during active maintenance of featural information.

The above findings are consistent with neural models that posit metric-dependent inhibitory interactions among items stored in VWM^12,13^. In particular, Johnson and colleagues^12^ proposed a dynamic field model that captured the processes of encoding, maintenance and comparison required in change detection VWM tasks see also^14^. In this model, inhibitory interactions between similar items produces a sharpening of the neural representation of each item, making it easier to detect subtle featural changes at test. These same inhibitory interactions also give rise to metric-dependent repulsion among similar items during active storage in VWM. In the present study, we report two new experiments that replicate previous findings of a repulsion bias during active storage in WM and we propose a novel neural dynamic field model and assess its ability to capture the observed pattern of results.

## Dynamic field model of working memory

To capture performance in recall tasks, we build on previous dynamic field (DF) models of VWM, change detection, and memory-based recall^12,14–16^. Within this framework, object surface features (e.g., specific orientations or colors) are modeled as localized activation peaks within populations of feature-tuned neurons (for similar formulations, see^13,17–20^). In the present case, inputs to the model are first registered in a low-level sensory field that encodes both the spatial and surface features of presented objects, in keeping with the coding properties of early visual areas. Localized peaks are formed when specific input causes activity within the population to cross a soft threshold that engages lateral interactions (self-excitation and surround inhibition). However, lateral interactions in the sensory field are relatively weak, and as a result, localized peaks only remain stable while input is present. This field provides input to a multi-layered feature WM system with lateral interactions tuned such that localized peaks can be sustained in the absence of continuing input. Neural populations exhibiting self-sustained activation of this sort have been identified in a range of different cortical areas, including the lateral prefrontal cortex, inferior temporal cortex, parietal cortex, higher order visual cortices, as well as subcortical nuclei including the basal ganglia (see reviews in^21,22^). Note, however, that it is unclear whether sustained activity in all of these areas is produced locally, or if it results from multi-regional interactions (see^23^ for consideration of local circuit and large-scale network mechanisms that could support sustained activation). Our model is agnostic on this issue. In any event, as more and more items are remembered, inhibition eventually becomes too strong to sustain an unlimited number of peaks. Consequently, only a small number of distinctive neural representations can be consolidated and maintained simultaneously in WM, placing an upper limit on capacity^13,14,19,24^.

In addition to supporting maintenance in WM, strong lateral interactions can also give rise to interactions among items when more than one item is stored. For example, when very similar items are stored (e.g., two nearly-identical colors), self-excitatory interactions associated with each peak can cause them to fuse into a single peak (see, e.g.,^13,14^). When peaks are a bit further apart, however, inhibitory interactions tend to dominate. In this case, overlapping surround inhibition from each peak produces a gradient of activation in which inhibition is strongest at field sites in-between each peak, and weaker on the outside edge (for evidence of surround inhibition in WM, see^25^). As a consequence, the peaks will tend to drift along the activation gradient towards field sites where activation is strongest—that is, away from each other. In the present study, we test this model prediction in a new experiment that is similar to the studies of repulsion bias described above^9,10^. Results replicated previous findings, confirming the predicted repulsion effect. Furthermore, probabilistic mixture modeling of response errors suggests that response variability (used as a proxy for mnemonic precision) differed as a function of the metric similarity of remembered items, with greater variability (lower precision) for more similar versus less similar items.

In the sections that follow, we provide a full description of the model architecture we used to capture these behavioral effects and illustrate its functioning through an example simulation. We then report the results of two behavioral experiments that replicate the finding of delay-dependent repulsion between metrically similar items actively maintained in VWM. Finally, we describe the results of two simulation experiments that provide quantitative fits of the observed behavioral results.

### Dynamic field architecture

The DF model captures patterns of neural activation, *u*, defined over relevant metric dimension, *x*, that evolve over time, *t*, in a manner described by a differential equation of the general form

1 $$ \tau \dot{u}\left( {x,t} \right) = - u\left( {x,t} \right) + h + s\left( {x,t} \right) + \int {k\left( {x - x^{\prime } } \right)} g\left( {u\left( {x^{\prime } ,t} \right)} \right)dx^{\prime } + q\xi \left( {x,t} \right) $$

where τ is a time constant, *h* is the field resting level, $s\left( {x,t} \right)$ is the external input to the field, and $\xi \left( {x,t} \right)$ is random noise scaled by noise level *q*. Lateral interactions in the field are defined by the convolution of the field output, $g\left( {u\left( {x,t} \right)} \right)$ (where *g* is a sigmoid function) with an interaction kernel, $k$. The interaction kernel describes connection weights as a function of distance in feature space. It is defined as a difference of Gaussians with local self-excitation and surround inhibition and, in some cases, a constant (global) inhibitory offset (e.g., in a field implementing attention functions). Dynamic interactions within and between fields promote the formation of localized peaks of activation, which form attractor states at the level of the neural population. In the case of sensory representations, these peaks provide stabilized detection of stimuli. In motor representations or attention control, competitive interactions between active regions produce selection decisions in which only a single activation peak can form even in the presence of multiple inputs. Finally, with sufficiently strong lateral interactions, activation peaks can become self-sustaining without external input and thereby serve as WM representations.

**Figure 1:**
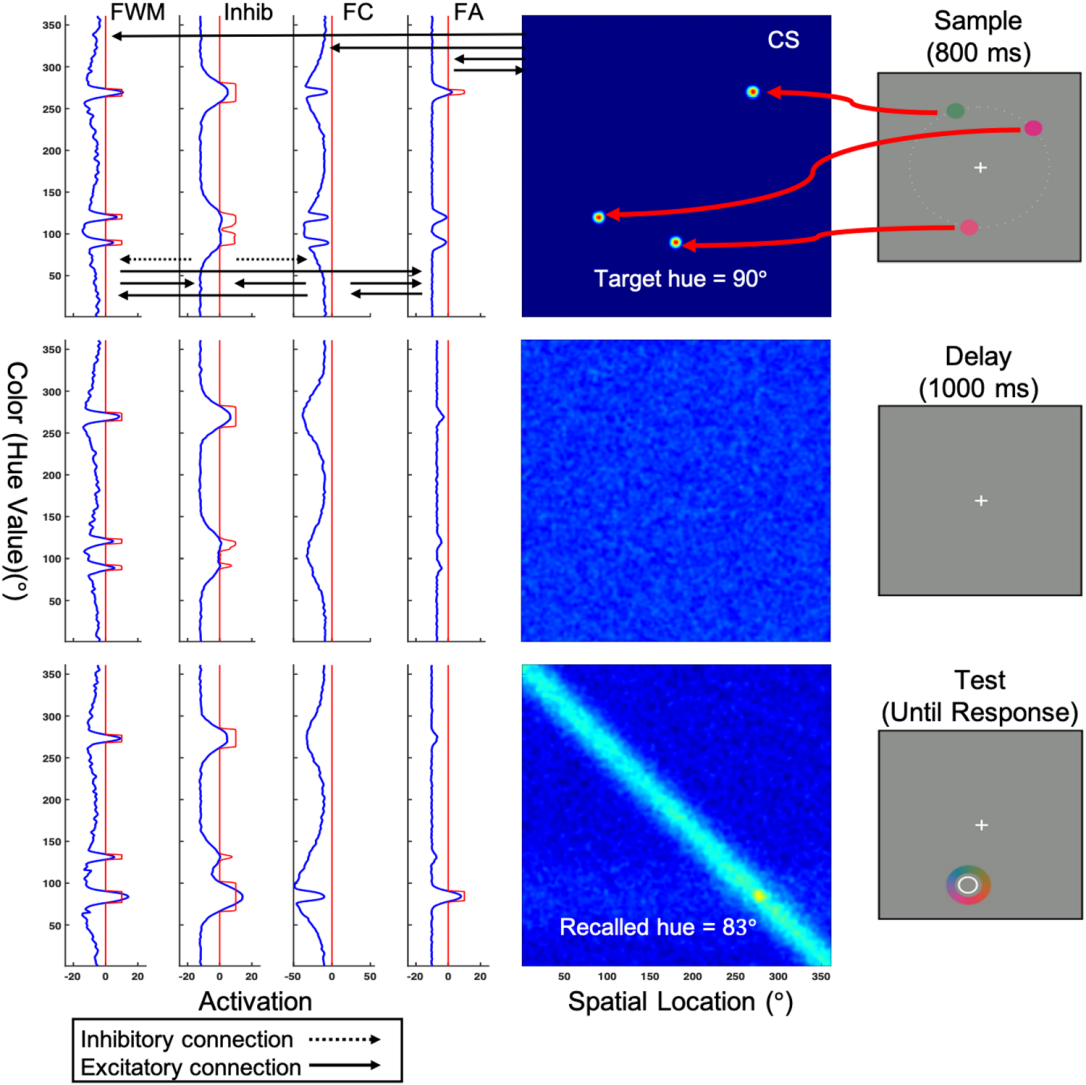
Model architecture used in Simulation Experiment 1 and a sample simulation of the model performing a single trial in the cued color recall task used in the behavioral experiment. The model consists of five fields: a two-dimensional visual sensory field defined over both color and space (CS), a feature attention (FA) field, a feature contrast (FC) field, an inhibitory field (Inhib), and a feature working memory (FWM) field, all of which are defined over the dimension of color. Blue lines in each panel represent the activation level (horizontal-axis) across field sites (vertical-axis), whereas the red lines depict the sigmoidal threshold function that determines the extent to which a given field site contributes to the activation dynamics. The task begins with the appearance of a sample display that contains either one or three colors. When three colors are presented, one of them is unique, and the other two are similar. Locally excitatory and laterally inhibitory interactions within and between fields support maintenance and produce repulsion between nearby peaks over the delay. See the text for full details of the model simulation shown here.

The model proposed here builds on the basic three-layer model of VWM originally proposed by Johnson and colleagues^12^, with two additional fields that allow the model to simulate performance in the cued color recall task commonly used to study VWM (see Fig. 1). The three-layer model was originally proposed to capture performance in the change detection WM task and consists of two excitatory fields reciprocally coupled to a single inhibitory field. The excitatory feature contrast field (denoted FC in the figure) is the main target of bottom-up input to the three-layer model, and the primary source of input to both the excitatory feature working memory (FWM) field and to the inhibitory field (Inhib). Locally excitatory interactions within the feature contrast field and FWM, together with broad inhibitory inputs from Inhib, allow localized peaks of activation to form in these fields in response to input. However, once input is removed, self-sustained peaks are only maintained in FWM, which features much stronger excitatory interactions than the feature contrast field. During the delay period, feedback from Inhib to the feature contrast layer, driven by input from FWM, produces localized regions of inhibition at field sites matching the features held in WM. This ensures that new peaks of activation will only form in response to inputs that are not currently being actively remembered. Thus, the feature contrast layer serves as a kind of novelty detector, which underlies the model’s ability to detect feature changes at test (as in^12^).

For the present work, two additional fields were added to the three-layer model. The first is a two-dimensional visual sensory field spanning the dimensions of spatial position (polar angle) and color (hue value). This field represents a simplification of the known properties of color-selective visual areas, in which a given neuron’s firing rate is determined by its coordinates in three dimensions; two dimensions of visual space and one dimension of hue (see, e.g.,^26–28^). The 2D Color-Space (CS) field is the primary target of sensory input to the five-layer model, and plays a critical role in allowing the model to make a spatially directed color recall response during the test phase of the task. Once input is turned on, activation initially builds in the visual sensory field and then propagates through a feature pathway that consists of the three-layer WM system described above with the addition of a feature attention field (FA). The feature attention field implements a weak form of feature-based competition that enables selection of specific inputs for encoding into WM, and, most critically in the present case, control of feedback input to the visual sensory field during recall. Each of the fields comprising the feature pathway are defined over the space of hue values. (Note that we assume the existence of a parallel spatial pathway that tracks the locations of remembered features [as in^16^]. However, for simplicity, we have elected not to include this pathway in the present model.) Excitatory and inhibitory connections between the model’s layers are indicated by solid and dashed arrows, respectively, as shown in the top panel of Fig. 1 (see Supplementary Materials for full model equations).

To facilitate understanding of how the various layers of the model work together to achieve multi-item WM and color recall, Fig. 1 shows a simulation of the model performing a single trial in the cued color recall task used here. At the beginning of the trial (topmost panel), three colored circles are presented at different spatial locations. This event leads to the formation of three localized peaks of activation in the 2D color-space sensory field (CS) representing the specific location and color of each stimulus in the task space. Above-threshold activation in the sensory field (integrated over the spatial dimension) is then propagated to the feature attention field (FA), the feature contrast field (FC), and, less strongly, to FWM. Direct excitatory input to the feature attention field leads to the formation of localized peaks representing the hue value of each stimulus. Lateral interactions within this field produce a weak form of feature-based competition in which each active region excites itself while suppressing other field sites. Above-threshold activation in the feature attention field combines with input from the sensory field to produce peaks of activation in the feature contrast field reflecting the novel hue values present in the task space. Although FWM also receives direct input from the sensory field, the primary input to this field comes from the feature contrast field, which is also the primary source of stimulus-related input to the inhibitory field. Stimulus input from the feature contrast field and the sensory field induces the parallel formation of localized peaks of activation in FWM, which is also reciprocally coupled to the inhibitory field. Once the memory display is removed (Fig. 1, middle panel), locally excitatory interactions within FWM together with strong surround inhibition from the inhibitory field allow sustained peaks of activation to be maintained in the absence of input. Additionally, inhibitory feedback from the inhibitory field to the feature contrast field, driven by sustained peaks in FWM, produces regions of inhibition in the feature contrast field corresponding to the stored hue values. Thus, new inputs that match the current contents of FWM are suppressed by the feature contrast field. As noted above, when used to capture performance in the change detection task, this plays a role in the ability of the model to detect featural changes at test. This field will also play a role in facilitating the sequential consolidation of memory display items in Simulation Experiment 2, described further below.

Another consequence of strong lateral interactions in FWM can be seen in the bottom panel of Fig. 1. Early in the delay period (middle panel), the peaks of activation in FWM are closely aligned with the position in hue space of the presented colors. However, by the end of the delay when the response wheel is presented (bottom panel), the activation peaks associated with the two similar colors have moved away from each other (i.e., have been repelled from each other) due to strong inhibition in-between versus on the “outside” of each peak. As a consequence, when one of these two items is subsequently reported at test, the estimated hue value is shifted away from the other nearby item in hue space.

To capture recall responses in the model (see Fig. 1, bottom panel), the appearance of the color wheel in the task space at test was modeled as a sub-threshold input along the diagonal of the 2D sensory field (spanning 360° of color and 360° of polar angle). The effect of the spatial cue was captured by a flat, local boost of the region surrounding the peak in FWM that matched the hue value associated with the cued spatial position. Here, we assume that this boost comes from attentional selection at the level of a scene representation that maps ‘what’ and ‘where’ and is reciprocally coupled to FWM, which is not explicitly modeled here (for an earlier implementation of this idea, see^29^). Note that this local boost was not precise. Indeed, it often robustly boosted one peak in FWM and partially boosted a neighboring peak.

The spatial cuing event increases the strength of an activation peak in FWM and biases activation in the feature attention field in favor of the cued item. We prompt the model to make a response by boosting the resting level in the feature attention field, which causes a peak to build at the field site representing the cued hue value and broad inhibition to suppress activity at field sites representing the other items in FWM. This peak, in turn, projects a horizontal ridge of activation into the color-space sensory field, boosting activity representing the remembered hue value across all spatial positions. A response peak then builds in the color-space field at the intersection of the diagonal ridge representing the colorwheel and the horizontal ridge reflecting color-specific input from the feature attention field. To derive a recall estimate, we then read off the spatial position of this peak and record the color value at that position in the wheel. This allows us to calculate a response error between the remembered hue value and the selected hue value associated with where the model ‘clicked’ on the color wheel. In Fig. 1, the response peak in the CS field is centered at 83° in hue space, whereas the target hue was presented at 90°. Thus, the model recalled the target hue as being seven degrees further away from the other close color than it actually was. That is, it produced a robust repulsion bias.

### Overview of the present study

In the present study, we first replicate the repulsion effect described in previous research using a task design optimized for simulation by the DF model described above. Specifically, the task requires the encoding and maintenance of a small set of colors drawn from a continuous color space and presented at particular spatial locations on an imaginary circle surrounding fixation. We then run a control experiment to rule out a perceptual account of the repulsion effects. Results confirm that a robust repulsion effect only arises in the case where a memory delay is inserted between the memory display and the cue to recall one of the remembered colors. In addition to the expected repulsion bias, we also observed metric-dependent differences in recall precision and a tendency towards increased guessing when the unique target item was cued at test. Specifically, recall precision was lower for two metrically similar colors versus a unique color stored at the same time. Additionally, the probability of storage of the unique item was somewhat lower than for either of the two similar items, contrary to what might have been expected based on studies of visual search in which unique items tend to “pop out” of the visual display see, e.g.,^30^. To our knowledge, these latter findings have not been reported previously.

We then ask if the DF model can quantitatively capture these findings in detail. An initial simulation experiment shows that the model captures the repulsion effect, but performs less accurately on precision and fails to capture the finding of reduced likelihood of storage for the unique item. Thus, we propose a modification to the model based on a recent model of scene representation that adds more details of a spatial pathway as well as implementing sequential consolidation in WM. Simulations reveal that this second model does a good job capturing all effects reported in the empirical findings including the details of the control experiment.

## Experiment 1

In the present experiment, we seek to replicate the finding of a repulsion bias between metrically similar colors using a procedure optimized for the model, which represents colored items in WM along a continuous 1D feature space. To do this, we use the procedure depicted in Fig. 1 to test the model prediction that metric-dependent interactions between items in FWM will lead to similarity-based feature repulsion. We then adopted a probabilistic mixture modeling approach^4^ to test for possible effects of metric similarity on putatively different sources of response error.

### Methods

#### Participants

Twelve neurologically normal college students (9 women; age range of 18–29) with normal or corrected-to-normal visual acuity and normal color vision participated in this experiment in exchange for monetary compensation. Each participant provided written informed consent and all procedures were approved by the University of Iowa Institutional Review Board. All methods were performed in accordance with relevant guidelines and regulations. Sample size was determined based on what were best practices in the lab at the time the study was run.

#### Materials

Stimulus presentation and response recording was controlled with a Pentium IV PC-based computer running custom software in Microsoft Visual Studio 6. Stimuli were presented against a gray background (28.73 cd/m^2^) on a 27″ Dell LCD monitor at a viewing distance of 70 cm. Sample arrays consisted of either 1 or 3 small (1.9° in diameter) colored circles. Individual colors were selected from a set of 180 colors equally distributed in CIELAB (1976) color space (centered at CIE L*a*b* coordinates: L = 70, A = 28, B = 12). Stimuli were presented on the circumference of an invisible circle with a radius of 7.5° positioned at the center of the screen, with a minimum distance between targets of 80° of angular rotation (see Fig. 1, top panel). For set size 1 (SS1) trials, a single color was randomly chosen from the 180 possible colors making up the color space. For set size 3 (SS3) trials, a single unique color was randomly chosen on each trial, then two similar targets were chosen that were 20° apart in color space—one clockwise (CW) and one counter-clockwise (CCW)—and 170° away from the unique target. The test array contained a color wheel with an outlined white circle at its center whose size and location matched one of the original sample array items (see Fig. 1C). The color wheel contained each of the 180 possible sample colors equally distributed in 2° steps. To prevent the adoption of a spatial strategy, the orientation of the color wheel was randomized on each trial and was presented equally often in either a standard or a mirror reversed fashion.

#### Procedure

Individual trials began with the appearance of a fixation cross at the center of the screen for 500 ms, followed by the 800-ms presentation of a sample array, a 1000-ms delay interval, and the appearance of a test display, which remained present until the participant made a response. Color recall responses were made by moving a set of crosshairs over the color wheel using a computer mouse, and left-clicking on the color that most closely matched the probed color (i.e., the color at whose location the color wheel was presented). The experiment was completed in one session consisting of a total of 800 experimental trials: 160 SS1 trials and 640 SS3 trials; 320 probing one of the two similar colors and 320 probing the unique color, randomly determined. Note that trial numbers for each condition were chosen so as not to bias participants towards remembering the two close items, which would be over-represented if trial numbers were equated across possible targets.

#### Statistical modeling of recall response distributions

To analyze performance in the recall task, response errors were calculated by subtracting each probed item’s correct value from the color value selected on the color wheel on a given trial. The resulting response error distributions were modeled as a mixture of a circular normal distribution and a uniform distribution using maximum likelihood estimation (as described in^4^). Model fits were separately obtained for each participant and each condition using the standard mixture model (with bias) implemented in the MemToolbox (v. 1.0.0)^31^. The model successfully converged for each participant. According to the logic of this approach, the mean (µ) and variance of the circular normal distribution capture the accuracy and resolution, respectively, of the representation when the probed item is present in memory at test. Conversely, the height of the uniform distribution gives an estimate of the probability that a given recall response was selected at random from the color space (P_uniform_). The parameter P_m_ can then be derived from the uniform distribution (1-P_uniform_) as an estimate of the overall probability that the probed item was present in memory at test (i.e., of WM capacity). Thus, this approach makes it possible to examine the impact of metric similarity on both the quantity and quality of information in WM. We should note, however, that mapping the separate parameters of the mixture model onto these putative sources of error relies on several underlying assumptions regarding the nature of storage in VWM that are still a matter of considerable debate in the literature (see^32,33^).

### Results

#### Analysis of mixture model fits

**Figure 2:**
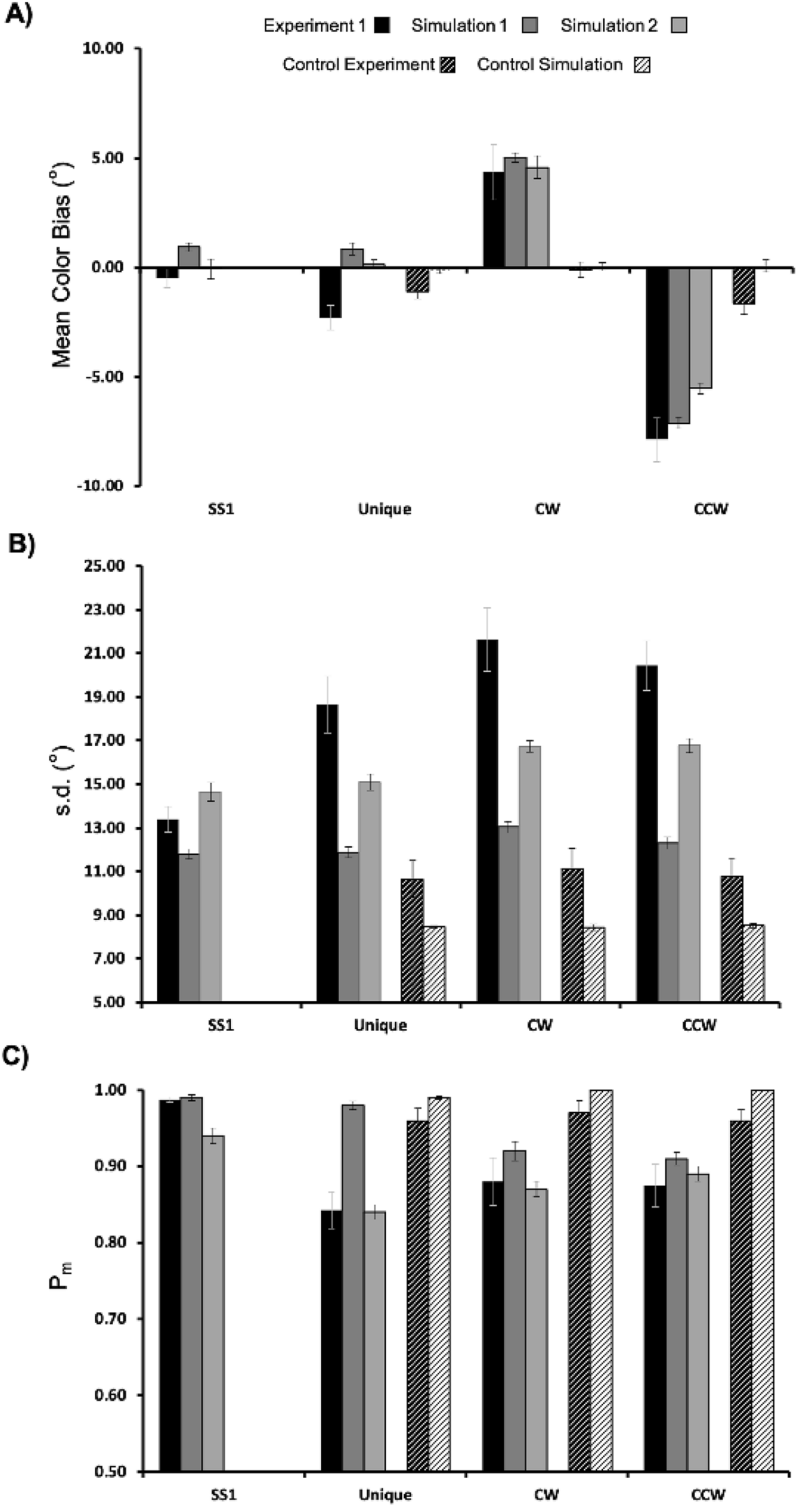
Comparison of mixture model fits derived from Experiment 1 subject data (black filled bars), simulation 1 (dark grey filled bars), and simulation 2 (medium grey filled bars) in the SS1 and SS3 conditions, and from the Control Experiment (white unfilled bars) and simulations (off white filled bars) for SS3 only. (**A**) Mean color error (μ) derived from the mixture model revealed a clear bias in color recall for each of the similar targets (CW and CCW), but only when a blank delay was imposed between the memory display and the test display, replicating previous findings. This effect was well captured by both variants of the DNF model. (**B**) Estimated mnemonic resolution (s.d.) also revealed an increase in variance in the SS3 versus SS1 condition, and higher s.d. for similar versus unique targets. This effect was moderately well captured by simulation 2 and less so for simulation 1. In each case, s.d. was lower in the simulations versus the subject data, and there were only small differences as a function of set-size. The resolution of color recall was lower overall in both the Control Experiment and simulations. (**C**) The likelihood that the cued item was stored in WM (P_m_) was higher in the SS1 versus SS3 condition overall in Experiment 1. In simulation 1, estimated P_m_ for the simulated data was elevated relative to estimates derived from the subject data. Simulation 2, which features sequential loading of items into WM, did a better job of capturing this effect in the data.

Individual parameter estimates averaged across participants are shown in Fig. 2A (black bars). (Note that model fits for each condition were also obtained for response distributions aggregated over participants, with very similar results. Data from these fits (using the swap model with bias) are reported in the Supplementary Results.) The accuracy of memory representations (reflected in the parameter µ) differed significantly across targets (SS1, Unique, CW, CCW), *F*(3,44) = 39.376, *p* = 1.60E-12, *η*^2^ = 0.73. Color estimates in the SS1 condition were highly accurate and were not significantly different from zero (*p* > 0.2), whereas estimates of the Unique target were biased in a negative direction, *t*(11) =  − 4.022, *p* = 0.001, d = 1.16. Critically, mean color estimates in the CW and CCW conditions significantly differed from zero (all *p*’s < 0.01), and response biases occurred in the predicted direction (i.e., positive in the CW condition, and negative in the CCW condition). That is, similar targets were repelled from one another as they were actively maintained in working memory, in keeping with previous results and the simulation depicted in Fig. 1.

Mnemonic resolution (s.d.) also varied significantly across targets (SS1, Unique, CW, CCW), *F*(3,44) = 27.13, *p* = 4.32E-10, *η*^2^ = 0.65. Colors were stored with higher resolution (i.e., s.d was smaller) when a single item was held in memory, versus three items, *t*(11) =  − 8.632, *p* = 3.15E-6, d = 2.49, in keeping with previous findings (see, e.g.,^4,34^). Additionally, unique colors were stored with higher resolution than were similar colors, *t*(11) = 2.47, *p* = 0.031, d = 0.71. This novel finding suggests that, with set size held constant, the resolution of individual items stored in working memory varies as a function of metric similarity.

Finally, the probability that the probed item was stored in memory (P_m_) also varied significantly across targets (SS1, Unique, CW, CCW), *F*(3,44) = 11.05, *p* = 1.564E-5, *η*^2^ = 0.43, with a greater likelihood of storage in the SS1 versus SS3 condition, *t*(11) = 5.06, *p* = 0.0004, d = 1.46. Contrary to what we would have expected, P_m_ was not significantly different for Close versus Unique targets, *p* > 0.2.

### Discussion

Results from the behavioral experiment confirmed previous findings of a repulsion bias for metrically similar features actively held in VWM (see^9,10^): metrically similar colors were remembered as being more distinct than they really were. Results also revealed an additional effect that, to the best of our knowledge, has not been previously reported in the literature: s.d. was found to be higher (resolution was lower) for metrically similar versus distinct colors held in WM at the same time. This finding is inconsistent with fixed resolution, slot-based views of WM (see, e.g.,^4^), and suggests that the resolution of individual items in WM is influenced by their metric similarity to the other items in WM. In addition to these effects, the likelihood of storing the Unique item throughout the delay (P_m_) was somewhat lower than it was for each of the two Close colors, although this effect was not significant. Given the well-established finding from studies of visual search that unique items tend to pop out of visual displays composed of otherwise homogeneous elements (see^30^), we expected the Unique item to be more salient than either of the two similar items, and therefore to be remembered more frequently.

The finding of a repulsion bias is consistent with initial simulations of the DF model of VWM depicted in Fig. 1, which demonstrates that, in the model, similar items held in WM interact in an inhibitory fashion that can give rise to memory biases. Before moving on to the simulation experiments, which attempt to quantitatively capture the repulsion effect and the other novel findings reported above, we first report the results of a control experiment aimed at ruling out an alternative, perceptual-level explanation for the observed repulsion bias.

## Control experiment

According to the DF model described above, the observed differences in mean error in the CW and CCW conditions arise as a result of strong inhibitory neural interactions between nearby peaks during active storage in WM. This causes the peaks to drift away from each other over the course of the memory delay. An alternative possibility, however, is that the observed effects represent a perceptual-level phenomenon in which similar colors either interact directly during perception, or are encoded together with information reflecting the categorical structure of the color space (see^35^ for evidence of categorical effects in WM for color). The observed bias could arise if the perception of similar colors is affected by perceptual-level color category effects that pull them in opposite directions. If this were the case, we would expect the repulsion effect to persist even when the memory delay is eliminated. This possibility was tested by Scotti and colleagues^10^, who found that a robust repulsion effect was only observed in a condition that featured a long delay between the memory display and the cue to report a given color. Here, we provide additional evidence against a perceptual locus for the repulsion bias in a control experiment that was identical to Experiment 1, with the exception that the color wheel appeared during the final 300 ms of the stimulus presentation interval. This design effectively reduced the length of the delay to be as close to zero as possible while preventing participants from simply ignoring the original display and focusing solely on the cued item at test.

### Methods

#### Participants

Twelve neurologically normal college students (8 women; age range of 18–33) with normal or corrected-to-normal visual acuity and normal color vision participated in this experiment in exchange for course credit. Each participant provided written informed consent and all procedures were approved by the North Dakota State University Institutional Review Board. All methods were performed in accordance with relevant guidelines and regulations.

#### Materials and procedure

Materials were identical to those described for Experiment 1, with the exception that stimulus presentation and response recording was controlled by a PC using custom code written in Matlab (Mathworks, Inc.) with Psychtoolbox extensions^36,37^, and stimuli were presented on the surface of a 24″ LCD monitor. Procedures were identical to those described above, with the exception that the color wheel and cue circle appeared 500 ms into the 800 ms presentation of the sample display, with both displays remaining on the screen together for an additional 300 ms. Additionally, the experiment only included the Unique, CW, and CCW conditions, resulting in a total of 640 trials (320 trials to the Unique target, and 160 each to the CW and CCW targets).

### Results

Parameter estimates averaged across all 12 subjects are shown in Fig. 2A (black bars with white cross-hatching). To assess the effect of the delay period on the separate parameters of the mixture model, we ran three separate, one-way ANOVAs comparing mean color bias (μ), P_m_, and s.d. across conditions. Mixture model fits for the parameter μ revealed a small negative bias in each condition (Unique =  − 1.10; CW =  − 0.11; CCW =  − 1.66). One-way ANOVA revealed that these differences were statistically significant: *F*(2, 22) = 8.18, *p* = 0.002, *η*^2^ = 0.43. Paired t-tests further revealed significantly greater negative bias in the CCW versus CW condition (*p* = 0.014) and in the Unique versus CW condition (*p* = 0.002), but not for the CCW versus Unique condition (*p* = 0.12). There were no significant differences as a function of color target for either s.d. (*F* = 1.12, *p* = 0.34) or P_m_ (*F* < 1, *p* = 0.67). The resolution of color WM was relatively high overall (s.d. was low: Unique = 10.66; CW = 11.15; CCW = 10.79; see Fig. 2, Panel B) compared to Experiment 1 (Unique = 18.66; CW = 21.63; CCW = 20.44), and P_m_ was generally very high across color targets (Unique = 0.96; CW = 0.97; CCW = 0.96; see Fig. 2, Panel C).

Although there were differences across conditions in terms of mean color bias, including a more strongly negative bias in the CCW versus CW condition, the magnitude of these effects was quite small, with a CW-CCW difference of less than 2 degrees. Additionally, mean color bias in the CW condition was very small and was also in a negative direction (mean color bias =  − 0.10), unlike the repulsion biases observed for both close color targets in Experiment 1 (see black bars in Fig. 2, Panel A).

Nonetheless, to more directly compare the results from Experiment 1 and the Control condition, we performed a mixed within- and between-subjects ANOVA with factors of Condition (Control, Experiment) and Color Target (Unique, CW, CCW). This analysis revealed a significant main effect of color target: *F*(2, 44) = 53.32, *p* < 0.0001, *η*^2^ = 0.50. The main effect of Condition was not significant (*F* = 1.74, *p* = 0.20). However, there was a significant Condition x Color Target interaction: *F*(2,44) = 31.85, *p* < 0.0001, *η*^2^ = 0.30. This reflects the fact that although mean bias for the Unique target was quite similar across experiments, there were large differences in mean error for the CW and CCW color targets in the Control Experiment versus Experiment 1.

### Discussion

As expected, recall performance was generally superior in the Control Experiment versus Experiment 1, which featured a 1-s blank delay between the memory display and the cue to recall a particular color. Specifically, memory display items were more likely to be successfully recalled at test (P_m_ was higher), were recalled with greater precision (s.d. was lower), and, importantly, mean color biases were lower overall compared to Experiment 1. The overall improvement in performance was not unexpected and can be readily explained in light of the model’s function. Briefly, in the presence of continuing stimulus input, peaks are higher amplitude, making them less susceptible to noise and stabilizing their position in the field near the actual target value. This reduces guessing by reducing the likelihood that a peak will fail before recall is complete, reduces s.d. by increasing the precision and the strength of input to the visual sensory field during recall, and reduces the impact of overlapping inhibition in-between versus on the outside edge of each close peak, which drives the repulsion bias. Essentially, the presence of continuing stimulus input at the field site representing the target color counteracts the tendency of the peak to move along its activation gradient. Once stimulus input is removed, self-sustained peaks are more susceptible to noise, are more likely to fail, and are more likely to drift along the activation gradient produced by overlapping inhibition between nearby peaks.

Most critically, this pattern of results conceptually replicates the findings of Scotti and colleagues^10^, who showed that robust repulsion biases in WM only arise with a sufficiently long delay between the presentation of the memory display and the appearance of the cue to report a particular color. Although mean color biases did differ significantly between the CW and CCW color target conditions in the Control Experiment, these differences were quite small (~ 1.5 degrees in color space) and did not occur in opposite directions in color space, like they did in Experiment 1. Thus, taken together, these results suggest that interactions occurring during perception can account for, at most, a small portion of the repulsion bias observed in Experiment 1. Instead, they suggest that metric-dependent repulsion between items arises during active maintenance of featural information throughout the memory delay. The ability of the DF model to capture these findings is explored in the following section.

## Simulation Experiment 1

The findings reported above are consistent with simulations of the DF model described above and shown in Fig. 1, specifically, that a repulsion bias arises as a result of similarity-based interactions among items during active storage in WM. In the current simulation experiment, we assess the ability of the DF model to account for the full pattern of similarity-based modulations of recall responses observed in Experiment 1 (see also^9,10^).

### Simulation procedures

The stimulus attributes and timings from the behavioral experiment were directly adapted for the simulation experiment (all model code is available at https://github.com/cosivina/cosivina_dft_projects). The model was run through the cued recall task as described in the example simulation and depicted in Fig. 1. The models’ recall responses on each trial were calculated by reading out the location along the spatial dimension of the CS field where above-threshold activation reached a local maximum. We then looked up the hue value presented at that position and response errors were estimated by calculating the difference between the selected hue value and the hue value of the actual target color.

The simulation experiment consisted of a total of 800 trials, as in Experiment 1: 160 SS1 trials and 640 SS3 trials (320 probing one of the two Close items and 320 probing the Unique item). Random noise was added to all field activations to obtain stochastic distributions of results. Full details of the procedure we used to tune the model parameters are given in the Supplementary Methods. Briefly, model parameters for the core model (FC, Inhib and FWM) were initially set to those used by Johnson and colleagues^12^ to capture metric-dependent differences in the context of change detection. Parameters for each of the additional fields were adapted from prior work and, when necessary, were adjusted by hand until a baseline level of stable model behavior was achieved. Once a set of parameters was identified, relevant parameter values were iteratively changed by hand across a series of simulation runs aimed at reducing the difference between the model’s output and that observed in the behavioral experiment described above. To simulate the performance of multiple participants, once a promising parameter set was identified, the full set of simulations was run 12 times, and estimates of mixture model parameters for each condition were derived from the recall responses generated on each run using the Standard Mixture Model (with bias) implemented in MemToolbox v. 1.0.0^31^. Like the behavioral results, reported simulation results therefore represent the mean (and standard error) of the model’s performance estimated across multiple runs (i.e., participants).

To quantify model performance, we computed the root mean squared error (RMSE) between mixture model fits derived from the model simulations and from the behavioral data (see Table 1). Additionally, to facilitate comparison of model fits between the two simulation experiments, we calculated the Akaike Information Criterion (AIC), which gives an estimate of goodness of fit while taking model complexity (i.e., number of free parameters) into account. The AIC was calculated using Gaussian Likelihood as follows: $${\text{N}}\log \left( {MSE} \right) + 2k$$, where N is the number of common data points simulated, MSE is the mean squared error (derived from the values given in Supplementary Table 1), and $$k $$ is the number of free parameters for each model. For the present simulations, the number of common data points simulated (N) was 12 (four conditions $$\times$$ three measures = 12 data points), and $$k$$ was 49 (details of how the number of free parameters were determined for each model are provided in the Supplementary Methods section).

**Table 1:** Root mean squared errors between simulations and empirical results.

	Mixture model parameters		
	Mean color bias (μ)	SD	P_m_
Simulation Exp 1	1.77	6.91	.07
Simulation Exp 2	1.70	3.61	.03
Control simulation	1.16	2.40	.03

### Results

The filled dark grey bars in each panel of Fig. 2 depict the estimated mixture model parameters derived from the first simulation experiment (referred to as *Simulation 1* in the figure). Estimated accuracy of simulated recall responses (referred to as *Mean Color Bias* in the figure) exhibited small positive errors in the SS1 and Unique conditions (Mean errors = 0.93 and 0.84, respectively), a large negative bias in the CCW condition (Mean error =  − 7.30) and somewhat smaller positive error in the CW condition (Mean error = 5.03). Estimates of mnemonic resolution (s.d.) for the simulated data (see Fig. 2B) revealed fairly low overall error (average s.d. across conditions = 12.22). Additionally, there was a small difference between s.d. in the SS1 versus the average of the SS3 conditions (Mean s.d. = 12.37 for SS3 and 11.78 for SS1). Finally, results for P_m_ (see Fig. 2C) revealed a high likelihood of storage in both the SS1 (M = 0.99) and Unique color (M = 0.98) conditions, and lower P_m_ for both CW and CCW Close colors (P_m_ CW = 0.92 in both conditions).

RMSE for the Mean Bias (μ) parameter was 1.77, with the largest difference between model performance and behavioral data in the Unique condition. RMSE for parameter s.d. (recall precision) was 6.91, with the largest discrepancy between model derived and behavioral estimates in the three SS3 conditions. Finally, RMSE for parameter P_m_, reflecting the likelihood that the target was successfully stored in WM, was 0.07, with the largest differences in the Unique condition. Overall AIC for the model used in Simulation Experiment 1 was 131.96. We will discuss this metric further when we compare the performance of this model to the model used in Simulation Experiment 2.

### Discussion

Simulations of the DF model of WM provided a solid fit to the empirical data. For the mean color bias effect (Fig. 2A), we expected errors in the SS1 and Unique conditions to be near zero, whereas, for the CW and CCW Close color conditions, we expected to observe pronounced biases in the opposite direction (positive for CW targets and negative for CCW targets). The model did a good job capturing this overall pattern, with small positive errors in the SS1 and Unique conditions, and a clear repulsion bias evident in the CW and CCW conditions.

For the s.d. effect (Fig. 2B), we expected variance in recall responses to be higher when three versus one item needed to be remembered (i.e., in the Unique, CW, and CCW conditions versus the SS1 condition), and higher for the two Close colors versus the Unique color. Both of these effects are expected to arise in the model as a result of increased variance in peak drift from trial to trial when multiple items are metrically similar. Although this general pattern was observed, s.d. across all conditions was much lower in the model than in the empirical data. Additionally, although s.d. was somewhat greater for CW and CCW targets than for targets in either the Unique or SS1 conditions, the effect wasn’t as pronounced as observed in the experiment, and s.d. was more or less identical in the SS1 and Unique conditions in the model, contrary to the experimental results.

With respect to the overall lower s.d. across conditions, part of the problem here lies in the process by which a peak in WM is translated into a response in the visual sensory (CS) field. Specifically, in this simplified model, the response is largely driven by featural input to CS, which does not require the selection of a spatial location, as in the task. It may be the case that noise would increase overall if a more realistic response mapping were implemented that included both spatial and featural inputs to the 2D visual sensory field following the cue. It is unclear, however, whether such a change would increase the size of the difference in s.d. between the SS1 and SS3 conditions.

Finally, with respect to P_m_, the model did a good job capturing the probability of storage in the SS1 and both close color conditions. However, simulations showed that the Unique item was successfully stored much more frequently in the model simulations than was observed in the empirical data. Indeed, there is a trend in the empirical data towards lower, rather than higher, P_m_ for the Unique versus Close color items. This feature of model performance is perhaps not too surprising given the parallel nature of memory encoding in the model. With relatively narrow spread of inhibition around each memory peak, the Unique item has little competition from the other items being encoded. As a consequence, the peak associated with this item tends to build more quickly and rarely fails to be consolidated. By contrast, the two close items compete with each other via shared lateral inhibition. This feature of lateral-inhibition type neural models has been used to capture the phenomenon of “pop out” mentioned above (see, e.g.,^38^). Given the ubiquity of this finding in studies of visual search, it is curious that, in the empirical data, P_m_ for the unique item was actually somewhat lower than for either of the two metrically similar colors.

One hypothesis that may explain the pattern observed in the empirical data is that P_m_ for the Unique item is lower because it tends to be consolidated in WM only after both of the Close colors have been consolidated. Although several studies have suggested that consolidation of simple features (color, direction of motion, orientation) can occur in parallel (see, e.g.,^39–43^), other evidence has suggested that when the stimulus display is presented for a sufficiently long duration, sequential consolidation is more likely^42^. The present paradigm featured a relatively long exposure duration compared to other studies (800 ms, compared to the more typical 100–500 ms durations used in other studies; see, e.g.,^4^). Thus, it is possible that participants attended to the similar colors and consolidated them first before consolidating the Unique item. This could have resulted in a failure to consolidate the Unique item on some trials and an increased likelihood of guessing (i.e. lower P_m_) when this item was probed at test. In our model, consolidation always happens in parallel so there is no way to capture this effect.

Interestingly, sequential consolidation is featured in a recent DF model of scene representation^29^. This model is similar to the model shown in Fig. 1, but it includes a multi-layer spatial pathway as well as an elaborated feature pathway that includes additional fields representing color and other object properties (e.g., orientation or size). Each of these pathways feeds into a series of two-dimensional fields that ‘bind’ features together into a scene representation. In particular, features at the same spatial location are linked together via cross-field excitatory interactions, implementing a distributed form of object representation mediated by location-based ‘binding’ (for evidence of location-based feature binding in WM and a novel neural model accounting for this effect, see^44^). To facilitate the accurate binding of object features at the scene level, the model implements a form of autonomous covert attention in which individual items are sequentially attended (as proposed by^30^ and others) and consolidated in WM one at a time.

In the simulation experiment that follows, we ask if we can adapt the model depicted in Fig. 1 and achieve a better fit to the data by implementing two aspects of the scene representation model: an explicit spatial pathway and sequential consolidation of items into WM.

## Simulation Experiment 2

**Figure 3:**
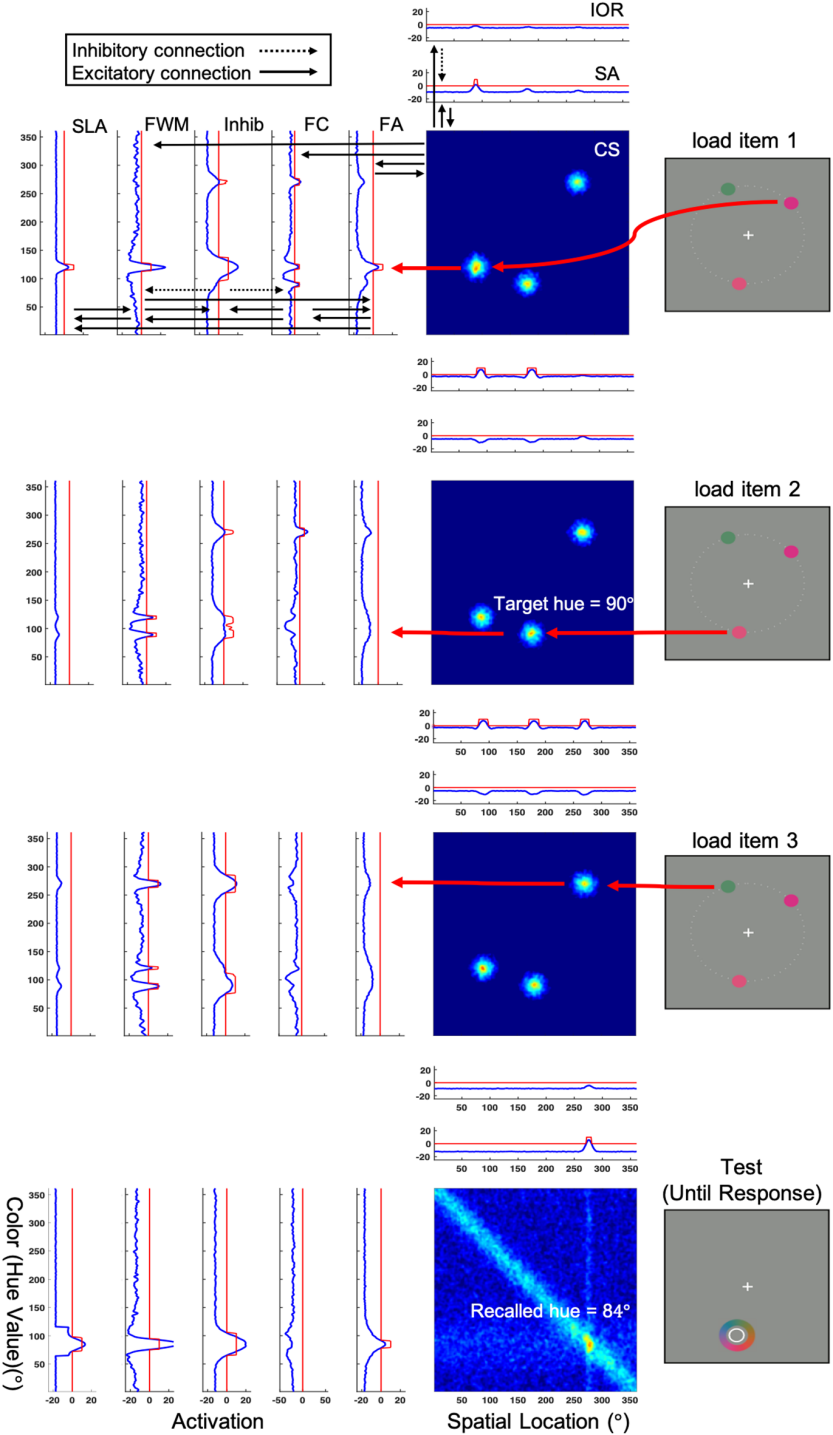
Model architecture and sample simulation of the simplified scene representation model used in Simulation Experiment 2 and the Control Simulations. This model includes an expanded spatial pathway that adds a spatial attention field (SA) and an Inhibition of Return (IOR) field, as well as a field representing attention at the scene level (SLA) to the model depicted in Fig. 1. These model additions give rise to an autonomous form of covert orienting that results in the sequential consolidation of items in WM, as shown in the top three panels of the figure. As before, blue lines represent the activation value across field sites, and red lines depict the sigmoidal threshold function. See the text for full details of the model simulation shown here.

The model used in the present simulation experiment is depicted in Fig. 3. This model incorporates aspects of the scene representation model described in^29^ including a simplified spatial pathway. The spatial pathway consists of a spatial attention (SA) field and an inhibition-of-return (IOR) field both of which are defined in the same retinal coordinates as the spatial dimension of the CS (color-space) field. The model also includes an additional one-dimensional attention field, defined in color coordinates, that stands in for attention operating at the scene level (SLA). Finally, the model includes a set of dynamic nodes: a peak detector (PD) node that detects the presence of an above-threshold peak of activation in the scene level attention field, and a condition-of-satisfaction (CoS) node that becomes active when the peak detector node goes above threshold and which plays a key role in the sequence of autonomous serial consolidation in FWM described below (note: for simplicity, these nodes are omitted from Fig. 3). Together, this simplified scene model tracks the spatial locations of remembered features and implements an autonomous form of covert attention that gives rise to sequential encoding of each item in the memory display. This modification turns out to be critical to capture the full pattern of results observed in the behavioral experiment. (see Supplementary Methods for full model equations, and Supplementary Tables 1–2 for model parameters).

A simulation of the modified model is shown in Fig. 3. As with the first model, when the memory input is turned on, peaks begin to form in the visual sensory (CS) field at the two-dimensional coordinates corresponding to the unique pairings of color and location defining each memory item. Feature-selective activation then propagates first to the feature contrast (FC) field, which detects the presence of novel items in the scene (i.e., items that are not currently being represented in WM), and then to the feature attention (FA) field. Activation along the spatial dimension of the CS field also propagates to the spatial attention field (SA). Competition among items then ensues in both attention fields, which operate in a winner-takes-all regime in the presence of strong global inhibition. Once an item gains a competitive advantage in one attention field, this same item becomes enhanced in the other attention field via coupling through the visual sensory field, implementing a form of biased competition (for a detailed account of this model feature, see^16^). The result is the selection of the color and spatial location of a single item from the memory display. This competitive advantage allows this item to be loaded into WM (i.e., for a peak to build in the FWM layer of the model) ahead of the other two items in the sample display (see topmost panel of Fig. 3).

After the first item is successfully consolidated, the sustained peak in FWM drives the formation of a peak in the scene level attention field. In the full scene representation model, this is where spatial and featural inputs would be bound together. Here, we simplify this picture and use the scene level attention field as a placeholder for the fully bound representation. This simplifies the spatial pathway as well as the scene level attention field, which is a two-dimensional (feature-space) field in the full model. Once a peak forms in the scene level attention field, this provides input to the peak detector node (not shown). Activation of the peak detector node signals that the item has been successfully ‘bound’ in scene level attention for the feature in question (color in this case). The peak detector node then activates the condition of satisfaction node (not shown), which boosts activation in the IOR field and suppresses activation in all three attention fields (FA, SA and SLA), triggering a disengagement from the current focus of attention. Now, with the first item successfully consolidated in FWM, activation associated with this item in the feature contrast field is inhibited by feedback from Inhib, signaling that the item is no longer novel. The remaining two items then compete for access to the focus of attention and the cycle repeats until each additional novel sensory input is consolidated. The sequential loading of each additional novel item into FWM is depicted in the second and third panels of Fig. 3. Once each item is consolidated, maintenance proceeds as in the model depicted in Fig. 1. For this reason, and for simplicity, the delay phase of the task was omitted from Fig. 3.

Finally, the generation of a recall response works similarly to the first model, with the exception that the cue to report one of the colors is generated by boosting activation in the scene level attention field, which then boosts activation around the cued item in FWM. In Simulation 1, this was achieved by boosting the item directly in FWM. The boost in activation surrounding the cued item in the scene level attention field and the increased amplitude peak at the corresponding location in FWM can be clearly seen in the bottom-most panel of Fig. 3. This gives rise to a peak in the feature attention field which projects a horizontal ridge of activation into the CS field. Once a peak begins to emerge at the intersection of the horizontal attention ridge and the color wheel input (see diagonal input in the bottom panel of Fig. 3), coupling with the spatial attention field causes a peak to emerge and the selection of a specific location on the color wheel. As with the first model, inhibitory interactions between the two nearby peaks in FWM leads to a repulsion bias: the response peak in the visual sensory (CS) field is centered at 84° in hue space, whereas the target hue was presented at 90°, an error of − 6° in color space.

### Simulation procedures

Simulation procedures were identical to those described for the first simulation experiment. Briefly, 160 simulated trials were run for the SS1, CW, and CCW conditions and 320 simulations were run for the Unique condition, with random noise added to each simulated run. Model parameters were iteratively modified across model runs in an attempt to match the behavioral data as closely as possible (see Supplemental Methods for full discussion of the parameter tuning procedure). Once a good set of parameters was arrived at, 12 runs of the model were completed, simulating the performance of different participants. All results reflect the average mixture model parameters across model runs, as in Simulation Experiment 1. Finally, the root mean squared error (RMSE; see Table 1) and the Akaike Information Criterion (AIC) were once again calculated as measures of model fit and to facilitate comparison of model performance between the two simulation experiments. For the present simulations, the number of common data points simulated was again 12 (four conditions $\times$ three measures = 12 data points), and $k$ was 56 (details of how the number of free parameters was determined for each model are provided in the Supplementary Methods section).

### Results

The filled light grey bars in each panel of Fig. 2 depict the estimated mixture model parameters derived from the second simulation experiment (referred to as *Simulation 2* in the figure). Estimated mean color bias in the model revealed near zero errors in both the SS1 and Unique conditions (Mean errors =  − 0.07 and 0.14, respectively). Additionally, there were prominent color biases in both the CW (Mean error = 4.46) and CCW (Mean error =  − 5.52) conditions. Critically, these biases were in opposite directions in color space. Estimates of mnemonic resolution (s.d.) for the simulated data (see Fig. 2B) revealed moderate overall variance in recall responses, compared to the behavioral data (average s.d. across conditions = 15.81 and 18.53 for the simulated vs. empirical data, respectively). Critically, there was an effect of set size on s.d., with greater s.d. in the SS3 (Mean s.d. across SS3 trial types = 16.19) versus the SS1 condition (Mean s.d. = 14.65). s.d. was also larger in the CW (s.d. = 16.73) and CCW (s.d. = 16.77) conditions than in the Unique condition (s.d. = 15.06). Finally, results for P_m_ (see Fig. 2C) revealed a high likelihood of storage in the SS1 condition (P_m_ = 0.94), lower P_m_ in the CW (P_m_ = 0.87) and CCW (P_m_ = 0.89) conditions, and lowest P_m_ in the Unique (P_m_ = 0.84) condition.

RMSE for the Mean Bias parameter was 1.70, with the largest difference between model performance and behavioral data in the Unique condition, followed by SS1 and the CW and CCW conditions. RMSE for parameter s.d. (recall precision) was 3.61, with the largest discrepancy between model derived and behavioral estimates in the three SS3 conditions. Finally, RMSE for parameter P_m_, reflecting the likelihood that the target was successfully stored in WM, was 0.03, with the largest differences in the Unique condition and the smallest difference in the SS1 condition. Overall AIC for Simulation Experiment 2 was 132.01.

### Discussion

The simplified scene representation model provided a good fit to the data from Experiment 1. In particular, the model captured the repulsion bias evident in both the CW and CCW close color conditions in Experiment 1, and more closely matched the pattern of s.d. across conditions, including lower overall s.d. compared to the SS1 condition and reduced resolution in the CW and CCW versus the Unique condition. Additionally, the simplified scene model did a better job capturing the qualitative pattern of reduced P_m_ for the Unique color observed in the behavioral experiment. Thus, although the AIC values were essentially identical across simulation experiments (AIC_Model1_ = 131.96; AIC_Model2_ = 132.01), it appears that the addition of an explicit spatial pathway and an IOR mechanism that implements sequential consolidation of memory display items allowed the model to better capture the overall pattern of recall errors suggested by the mixture model analysis. In the section that follows, we assess whether the simplified scene model can also capture the pattern of findings from the Control Experiment.

### Control experiment simulation

In addition to capturing the pattern of results observed in Experiment 1, we also conducted a set of simulations aimed at using the simplified scene representation model depicted in Fig. 3 to capture performance in the Control Experiment. Getting the model to perform the control task, in which the stimulus display and the colorwheel/recall cue overlapped in time for 300 ms, required a few modifications. In the experiment, there is a shift in the spatial frame of reference from the full display area to the region of the screen where the colorwheel is presented (see task schematic in Fig. 1). In effect, participants ‘zoom in’ on the colorwheel at test. This change in reference frame is not explicitly modeled; instead, we change the spatial frame implicitly during the transition from the memory delay to the response phase of the task. This works fine when the memory display and the colorwheel are separated in time, but creates a clash of reference frames when they are presented together. For the control experiment, our assumption is that, during the response phase of the task, participants’ attention shifts to the colorwheel, the cue, and the cued color, and away from the other items in the display. As a result, the uncued colors fade into the background as people focus on the relevant region of space. To model this, we reduced the strength of the two uncued inputs during the 300 ms interval when they are presented together with the colorwheel. We also modified the spatial layout of the memory display items so they would not overlap with the diagonal ridge representing the colorwheel. With these modifications, the model is able to perform the recall task with the same timings used in the control experiment. Besides these modifications, all model parameters were held constant and simulation procedures were identical to those used to capture performance in Experiment 1.

## Results and discussion

Mixture model fits for the Control Experiment Simulation can be seen in Fig. 2 (white bars with black cross-hatching). There were a few small discrepancies between the model’s performance and the behavioral results: mean color biases were flatter across targets, s.d. was lower (resolution was higher), and P_m_ was slightly higher than in the behavioral data. Despite these differences, the model did a good job overall capturing the pattern of results observed in the Control Experiment. Critically, these simulations show that, in the model as well as the behavioral data, a sufficiently long delay between the stimulus display and the response phase of the task is required to observe a robust repulsion bias.

### General discussion

The present study aimed to replicate recent reports of a repulsion effect between similar features stored concurrently in WM and to capture this phenomenon using a novel DF model of WM. To do this, we conducted two separate color recall experiments that differed only in whether there was a blank delay inserted in-between an initial stimulus display and a subsequent colorwheel and cue to recall a particular remembered target. We also described two novel DF models of WM, which differed in their complexity, and used them to simulate performance in the behavioral tasks.

Results from Experiment 1 replicated the repulsion effect observed in recent experiments and predicted by the model simulations depicted in Fig. 1, and revealed an additional novel result that, to the best of our knowledge, has not been previously reported. Specifically, results confirmed that recall estimates for similar colors are biased away from each other during active storage in WM. In keeping with this possibility, results from the Control Experiment, in which the memory delay was eliminated, revealed significantly reduced memory biases compared to Experiment 1. In particular, although there was a small but significant repulsion bias between the CW and CCW color targets in the Control experiment, this effect was nearly an order of magnitude smaller than the repulsion effect observed in Experiment 1 (1.55 vs. 12.23 degrees; this difference is comparable to the results of Scotti et al.^10^ Experiment 1 versus Experiment 2). Additionally, both Close targets in the Control experiment were weakly biased in a negative direction (CW =  − 0.10 and CCW =  − 1.66). Given this, we contend that the repulsion effect observed in Experiment 1 largely depends on the same dynamic neural processes that mediate active storage and limit the number of distinct items that can be simultaneously stored in WM. In the model, this effect arises as a result of strong inhibitory interactions between nearby peaks in WM, which are stronger at field sites in-between versus on the ‘outer’ edge of each peak. The inhibitory gradient established by these interactions causes each peak to move along the activation gradient until inhibition is balanced on either side of each peak. Observing this phenomenon requires a sufficiently long delay between the stimulus display and the recall test.

Behavioral results also revealed a metric-dependent decrease in the resolution of color WM (s.d.), which, to the best of our knowledge, has not been previously reported. Specifically, mixture model fits suggested that unique colors were stored with higher precision than were metrically similar colors stored at the same time. If real, this finding violates the claim made by proponents of the discrete slots view (see, e.g.,^4^) that WM stores a small number of items in independent storage slots at a fixed level of resolution. However, it is possible that this result could have been an artifact of the mixture modeling procedure, rather than a real effect of metric similarity on recall precision. For example, recall precision for the two close items could have been spuriously elevated if the mixture model had difficulty attributing a given reported feature to the correct distribution on close color trials. This could have arisen if the participant mistakenly reported the un-cued (aka “non-target”) close feature, instead of the cued close color target, on some portion of trials. This type of “swap” error is known to occur with some frequency in studies of VWM (see, e.g.,^34,45^). Although this certainly could have occurred on some trials, if it were happening with sufficient frequency to account for the 2–3 degree increase in s.d. observed in the Close versus Unique color conditions, we would have expected mean errors to be biased *towards*, rather than *away from*, the close non-target color.

Nonetheless, to examine this possibility further, we conducted a separate mixture model analysis using the three-component “swap” model proposed by Bays and colleagues^34,45^. This is reported in full in the Supplementary Results section. Briefly, this analysis revealed that the probability of non-target responses was elevated in the Close versus Unique color conditions, with non-target responses on Close color trials (i.e., on trials when either the CW or CCW target was cued) clearly clustering around the location in color space of the Unique non-target (see response distributions in Supplementary Figure S1A-C). However, there was no obvious clustering of responses around the close non-target item. Instead, responses were more likely to cluster in the four or five bins moving away from, rather than towards, the other close item, as would be expected given the finding of an overall repulsion bias.

Another possibility is that the finding of increased s.d. in the Close color conditions may simply reflect a trade-off between s.d. and guess rate (g), which are known to be correlated in some cases (see discussion in^31^). Recall that s.d. was greater and g was lower (P_m_ was greater) in the Close versus Unique color conditions. To examine whether the increase in s.d. could be explained by a trade-off of this sort, the Supplementary Results section also includes heatmaps depicting the relationship between model estimates of g and s.d. for each condition (see Supplementary Results, Figure S2). These plots reveal a negative correlation between these parameters, which suggests that the data in each condition are equally consistent with a slightly higher g and a slightly lower s.d., or with a slightly lower g and higher s.d. However, as can be seen in the separate panels of Figure S1, the credible ranges given for these parameters for the Close versus Unique conditions are entirely non-overlapping. Thus, this difference would persist even if one or both parameters was closer to the edge of its credible range, suggesting that this effect likely cannot be explained as a simple trade-off between these parameters.

Taken together, the results summarized above, and described more fully in the Supplementary Results, increase our confidence that the observed increase in s.d. in the Close color conditions likely reflects a real effect, rather than an artifact of the mixture modeling procedure. This finding, and the ability of both DF models to capture it, is consistent with the suggestion that individual items stored in WM interact, and the metric-dependent nature of this interaction gives rise to both the repulsion effect described above, as well as an increase in recall variance. In the model, reduced resolution for highly similar colors likely arises as peaks move along the activation gradient produced by inhibition from the other peak. This produces a systematic bias as well as an increase in the variance of estimated peak position across trials. By contrast, the unique item is less likely to be influenced by the presence of strong activation gradients (i.e., to drift over time), and therefore recall estimates for this item are generally more stable across trials.

In addition to capturing these effects, both models exhibited an overall increase in guessing (i.e., reduced P_m_) and a reduction in resolution (higher s.d.) as a function of set size (SS3 versus SS1; for similar findings, see^4^), although overall s.d. and the effect of set-size on this parameter were smaller than observed in Experiment 1. In the model, guessing typically occurs when a stable representation of one of the memory display items (i.e., a memory peak) fails to build in WM, or one or more of the peaks in WM is destabilized prior to the generation of a recall estimate. Such destabilization can occur when inhibition from other items in WM effectively suppresses the recurrent excitatory activity necessary to maintain stable peaks. Similarly, increased recall variance in the SS3 condition could be due to an increase in peak movement caused by the presence of other items in WM. This effect is most pronounced for each of the close items, but, as seen in Fig. 2B, s.d. is also increased to a small extent for the unique item, relative to SS1. Thus, the DF model provides a parsimonious neurodynamic account of the commonly observed positive relationship between guessing (P_m_), memory resolution and set size. By contrast, Zhang and Luck^4^ proposed to capture set-size effects on resolution via an ad hoc assumption that, for trials in which the set size is less than the number of available slots, separate slots can be used to store independent samples of the same stimulus. The average of these samples is then reported at test, resulting in improved resolution.

Although the model used for Simulation Experiment 1 did a reasonably good job capturing the overall effect of set-size on P_m_, it failed to capture the differential pattern of P_m_ across the unique and similar items in WM. As can be seen in Fig. 2C (black filled bars), participants exhibited a general decrease in P_m_ that was comparable for all three items stored. By contrast, the model closely matched P_m_ for the two close color targets, but overestimated P_m_ for the unique target. This difference may be explained as a result of local inhibitory interactions in the model, which have a substantially larger effect on nearby versus more distal peaks. Specifically, although the unique target is affected by moderate global inhibition produced by the other two items in WM, each of the close peaks receives strong inhibition from the other nearby peak. In some cases, this is sufficient to suppress the excitatory activation necessary for the peak to be sustained throughout the delay, resulting in elevated guess-like responses (i.e., lower P_m_).

The poor performance of the first model in capturing the pattern of P_m_ described above and the relatively low s.d. across conditions compared to Experiment 1, motivated the development of the elaborated model shown in Fig. 3. We hypothesized that lower P_m_ for the unique color target could have arisen as a result of sequential consolidation of the display items into WM, and a tendency to focus first on the two close colors. To examine this possibility in the model, we adopted a simplified version of a scene representation model (see^29^) whose autonomous dynamics give rise to sequential consolidation of memory display items. Interestingly, in this model, the two close items tend to be consolidated before the unique item. This arises as a result of overlapping inputs from the visual sensory field to the feature attention field for the two close colors, which pushes activation in this region of the color space closer to threshold. This can be seen in the feature attention (FA) field of the first few panels of Fig. 3. Overlapping inputs for the two close colors in feature attention creates a bias for peaks to build here first, and in turn, for activation related to the close colors to propagate to the feature contrast and FWM fields, where consolidation occurs. This feature of the model increases the likelihood that the unique color will fail to be consolidated in FWM, allowing the model to capture the qualitative pattern of lower P_m_ for all targets in the SS3 versus SS1 condition. Additionally, this model more closely matched the overall magnitude and pattern of s.d. observed across conditions, and did a good job capturing the pattern of findings observed in the Control Experiment. Critically, mean color biases in the model were near zero for each target. Additionally, s.d. was reduced and P_m_ was increased compared to Experiment 1 and Simulation Experiment 2.

Although the DF model did a good job capturing the main experimental findings from Experiment 1 and the Control condition, there were a few curious aspects of the results that are not very well understood. Specifically, there was an overall tendency for recall estimates to be biased in a negative direction in the SS3 conditions. This is most clearly demonstrated by the finding of a significant negative bias for the Unique color in Experiment 1 and, to a lesser extent, in the Control Experiment. There was also an asymmetry in the repulsion effects observed in the CW and CCW color conditions of both experiments, with a large negative bias in the CCW condition and a smaller positive bias in the CW condition. These small effects were not predicted by the model, and it is not clear what caused them. One possibility we considered was that all of the display items were being drawn towards the ensemble average color of the memory display, which has been observed to occur in other studies of multi-item VWM (see, e.g.,^5–8^). However, it isn’t clear why this would produce the small negative bias observed here, given that the Unique item was always exactly 170 degrees away from each of the close color targets in color space. Whatever the cause, these effects are secondary to the main findings of a repulsion bias and increased s.d. for metrically similar colors.

In summary, both models reported here provide a neurally plausible account of similarity-based repulsion in WM and the dependence of this effect on the presence of a memory delay. Although both models produced comparable fits to the observed data, as indicated by their near-identical AIC values, the second model accurately captured both the pattern of reduced P_m_ in the Unique versus Close color conditions as well as performance in the Control Experiment. Taken together, we think this argues in favor of Model 2, which implements a form of autonomous sequential consolidation in WM.

### Relationship of dynamic field model to other prominent models of WM

The proposed neurodynamical model builds on previous models that have aimed to capture working memory, attention, and related phenomena using attractor dynamics within populations of location and feature-selective neurons. Although the goal here is to show how neural population dynamics can give rise to and support perceptual and cognitive phenomena, this model shares some properties with other prominent frameworks for thinking about WM. For example, the dynamic neural processes underlying maintenance in the model impart a discrete, “all-or-none” quality to neural representations and can give rise to capacity limits at higher set sizes (see, e.g.,^14,15^). This reflects the bi-stability underlying the peak state—peaks either form or they do not. In this sense, WM peaks in the model could be thought of as the neural implementation of a discrete, slot-like form of WM postulated by Zhang and Luck^4^ and others (see, e.g.,^46,47^). However, the results reported here (see also^12^) suggest that items in WM interact in particular ways depending on their metric similarity. Additionally, previous findings within this framework suggest that capacity may not be fixed across trials, but likely varies dynamically as a function of noise as well as metric similarity^15^, a finding that likely also applies to mnemonic resolution (see, e.g.,^48^). Both of these properties set this framework apart from standard slot-based views.

In addition to sharing some commonalities with slot-based approaches, the proposed model is similar to a recent hybrid slot/resource model of working memory proposed by Wei and colleagues^13^ (see also^18,19^). Like the DF model, maintenance in the Wei et al. model is achieved by interactions between separate populations of excitatory and inhibitory feature-tuned neurons (i.e., locally excitatory and surround inhibitory interactions). However, their model is implemented in a more biophysically realistic framework (spiking network versus abstract population code model), and interactions between nearby items in WM are primarily excitatory in nature. As a consequence, WM peaks representing very similar features have a tendency to merge into a single peak over the memory delay, which provides one source of capacity limits in their model. Although mutually excitatory interactions do come into play in the DF model when colors are very similar, at more intermediate separations, such as probed here, inhibition tends to dominate. Such inhibitory competition can contribute to peak failure, and, importantly, can also account for the finding of similarity-based feature repulsion reported here and elsewhere^9,10^. It is unclear how this effect could be accounted for in the Wei et al. model.

Although the lateral-inhibition type model proposed here provides a plausible account of repulsion biases observed in some WM tasks, there are a number of challenges that it would be beneficial to address in future research. First, there may be other neurally plausible approaches that could similarly capture, or predict, these and related phenomena, and such approaches should be developed and tested. For example, Bays and colleagues^49–51^ have proposed a population coding approach in which errors in WM tasks are accounted for by noise in feature-tuned neural populations (for a related approach pitched at a different level of analysis, see^52^). Within this framework, informative cues about which item(s) are relevant on a given trial increase the gain of neurons coding for the relevant features, at the expense of task-irrelevant information. This framework has done a good job to date explaining memory errors in a neurally plausible framework, but it is unclear whether it could provide a principled account of the kinds of memory distortions examined here.

In addition to testing the DF model against other neural models, an important step in the process of validating the models proposed here will be to generate and test novel predictions in new experiments. Although this important goal is beyond the scope of the present paper, a way forward is suggested by previous research within this framework looking at delay and experience-dependent distortions in spatial WM (see, e.g.,^53–57^). For example, work in the spatial domain has shown that recall of the location of a single stimulus held in WM is biased away from salient axes of reference (e.g., the midline of the task space) over the memory delay (see^57,58^). Interestingly, in other contexts, this midline repulsion effect is observed together with an attraction effect, in which recall of the location of a single stimulus is biased towards the location of a frequently viewed target (see^55^). Both of these findings can be captured by a DF model that incorporates a Hebbian learning mechanism that allows traces of items viewed and held in WM to accumulate over time, producing peak drift in the direction of frequently viewed targets. Although preliminary, recent evidence from a similar study involving WM for color suggests that recall of individual colors may also be influenced by the distribution of color targets that were remembered throughout the experiment^59^. Work of this sort could provide a principled account of the conditions under which different kinds of memory distortions of the sort described in^11^ may be expected to arise.

In conclusion, results confirm the presence of a repulsion effect in WM when similar features are held actively in mind over a memory delay. This phenomenon was captured by a neurodynamical model in which items in WM interact in a metric-dependent fashion and give rise to characteristic behavioral signatures that are evident in performance. Taken together with previous findings, the current results suggest that this neurally-grounded framework offers insights into both the neural population dynamics that underlie working memory for simple visual features and the behavioral signatures evident in experiment that are critical to testing competing theories of working memory.

## Supplementary Information

Supplementary Figure 1.

Supplementary Figure 2.

Supplementary Information 3.

Supplementary Table 1.

Supplementary Table 2.

Supplementary Table 3.

## Data Availability

The datasets generated and analyzed as part of the current study are available at the following URL: https://github.com/cosivina/cosivina_dft_projects/tree/master/Johnson_ScientificReports_2022.
