# Supplementary figures and images for: Neural interactions in working memory explain decreased recall precision and similarity-based feature repulsion
Source: Sci Rep. 2022 Oct 22;12:17756. doi: 10.1038/s41598-022-22328-4 (PMC9588047; doi:10.1038/s41598-022-22328-4)

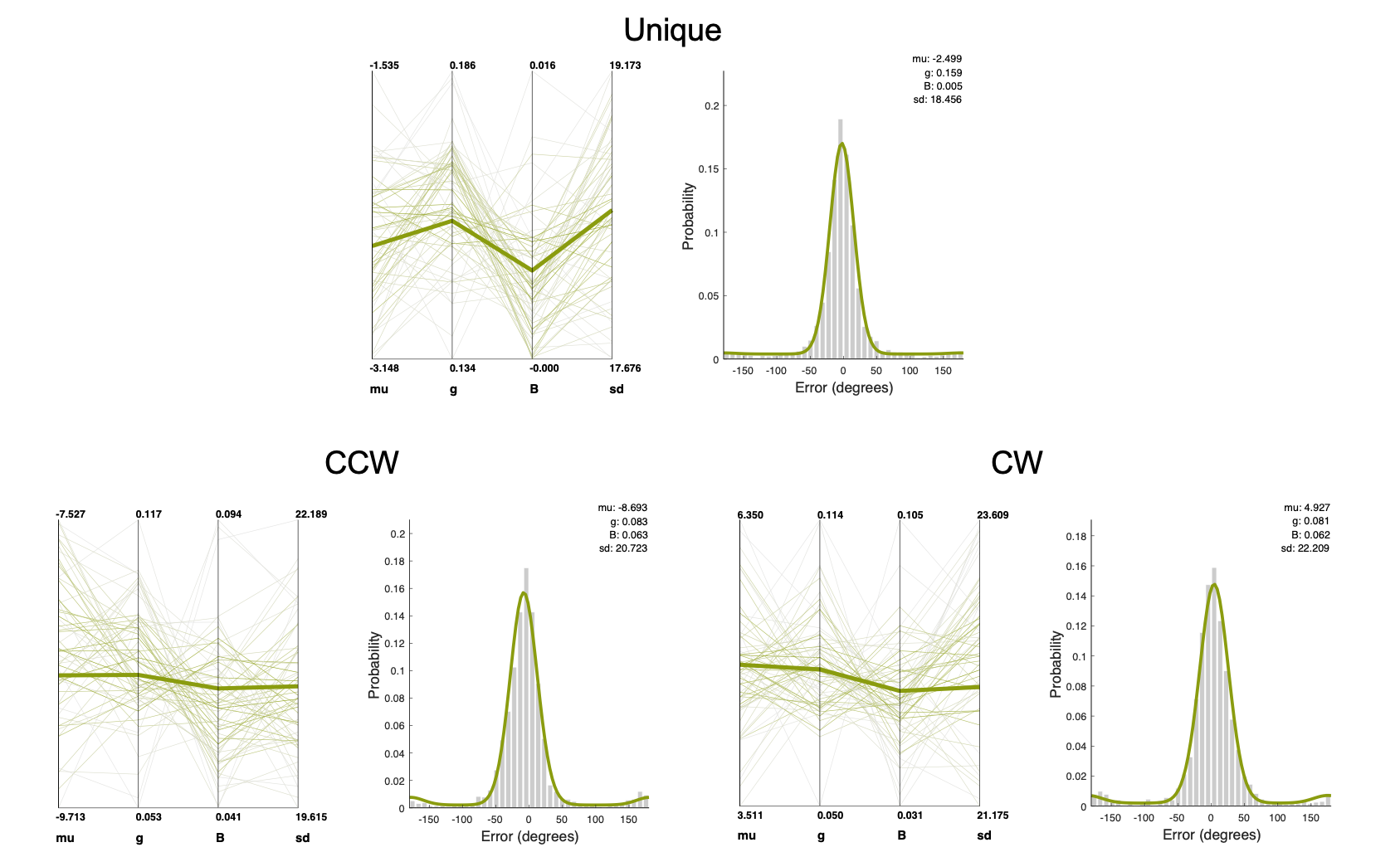

Supplement: Supplementary file 1 — Supplementary Figure 1. [file 41598_2022_22328_MOESM1_ESM.png]

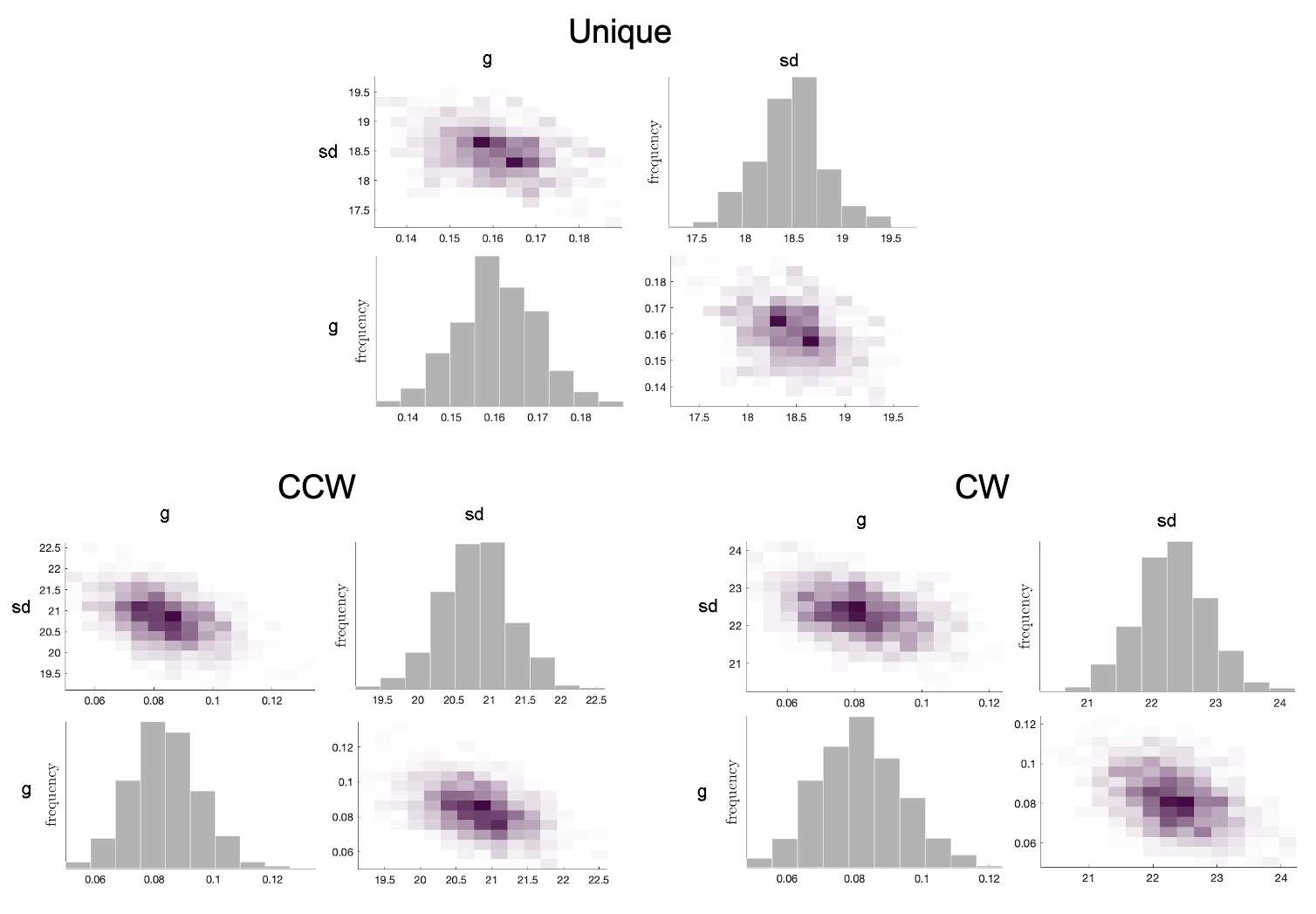

Supplement: Supplementary file 2 — Supplementary Figure 2. [file 41598_2022_22328_MOESM2_ESM.png]
