# Supplementary material for: Neural interactions in working memory explain decreased recall precision and similarity-based feature repulsion
Source: Sci Rep. 2022 Oct 22;12:17756. doi: 10.1038/s41598-022-22328-4 (PMC9588047; doi:10.1038/s41598-022-22328-4)
Supplement: Supplementary file 3 — Supplementary Information 3. [file 41598_2022_22328_MOESM3_ESM.docx]

**Supplementary Methods: Neurodynamical Models and Simulation Procedures**

**Model 1 architecture and simulation procedures**

The model depicted in Figure 1 and used in Simulation Experiment 1 constitutes an integrated dynamical system composed of 5 DFs, four of which span a single dimension (color, *y*, in this case), and one of which spans the two dimensions of color and space (*x*). In the equations below, we identify each field by a unique index: *v* for the two-dimensional visual sensory field, *fa* for feature attention field, *fc* for feature contrast field, *fi* for feature inhibition field, and *fwm* for feature working memory field. Parameters of projections between fields are identified by two indices, the first indicating the target, the second the source of the projection.

For numeric simulations, the fields were sampled at discrete, equidistant points and the activation values were updated in fixed time steps of 2 ms using the Euler method. Time constants and additional parameters for each field equation underlying Model 1 are given in Supplementary Table 1 and Supplementary Table 2. Color and spatial dimensions were sampled with 361 units and were defined in a circular manner.

Activation within the two-dimensional visual sensory field is governed by the field equation

where τ is a time constant (note that time is omitted from two-dimensional equations for simplicity), *h* is the field resting level, $s\left(x,y\right)$is the external input to the field, and *noise* is spatially correlated noise. Lateral interactions in the field are defined by the convolution of the field output $g\left(u\left(x,y\right)\right)$ with an interaction kernel *k*. The output of all fields is computed from the field activation, *u*, via the sigmoidal function: $g\left(u\right)=\frac{1}{1+\exp\left[-\beta_{u}u\right]}$ with steepness parameter *β*. The steepness parameter determines the degree to which neurons close to threshold (i.e., 0) contribute to the activation dynamics. Lower values permit graded activation near threshold to influence performance, whereas higher values ensure that only above-threshold activation contributes to the activation dynamics. For the visual sensory field, the interaction kernel is described as a difference of two Gaussians, a narrow excitatory component and a wider inhibitory component, and a global inhibition term:

$$k\left(x,y\right)=c_{exc}\cdot\exp\left[-\frac{1}{2}\left(\frac{x^{2}}{\sigma_{x,exc}^{2}}+\frac{y^{2}}{\sigma_{y,exc}^{2}}\right)\right]-c_{inh}\cdot\exp\left[-\frac{1}{2}\left(\frac{x^{2}}{\sigma_{x,inh}^{2}}+\frac{y^{2}}{\sigma_{y,inh}^{2}}\right)\right]-c_{gi}$$

Here, $c_{exc}$is the strength of the lateral excitation, and $\sigma_{x,exc}$and $\sigma_{y,exc}$are the width parameters along each dimension. Although the width parameters can be set independently for each dimension--for example, they may be broad along one dimension and sharp along the other-- for the present simulations, they were chosen to be identical. The parameters $c_{inh}$, $\sigma_{x,inh}$, and $\sigma_{y,inh}$analogously describe the inhibitory Gaussian component, and $c_{gi}$is the strength of global inhibition.

In addition to lateral interactions, activation is further modulated by excitatory feedback input from the feature attention field, smoothed by an interaction kernel. The interaction kernels for all one-dimensional projections take the form of a Guassian with a single excitatory component:

$$k\left(y,t\right)=c_{exc}\cdot\exp\left[-\frac{1}{2}\left(\frac{y^{2}}{\sigma_{y}^{2}}\right)\right]$$

as with the two-dimensional field, $c_{exc}$ sets the strength, and $\sigma_{y}$sets the width of the projection.

Finally, activation in the field is influenced by the addition of a stochastic component consisting of spatially correlated noise,

$$noise\left( x, y,t \right)= c_{noise}\iint k_{noise}\left( x-x^{'},y-y^{'} \right)\xi\left( x^{'},y^{'} \right)dx'dy'$$

Noise was added to the simulations by convolving a noise field composed of independent noise sources with a Gaussian kernel specified by:

$$k_{noise}\left( x-x^{'},y-y' \right)=\frac{1}{\sqrt{{2\sigma}_{noise}}}exp\left[ -\left( x-x^{'},y-y' \right)\frac{2}{{2\sigma}_{{noise}^{2}}} \right]$$

where $\sigma_{noise}$ is the spatial spread of the noise kernel.

External sample inputs to the field, $s\left(x,y\right)$, took the form of a Gaussian in two dimensions. For each localized stimulus at a field location $\left[p_{x},p_{y}\right]$, the input was given as:

$$s\left(x,y\right)=a_{s}\cdot\exp\left[-\frac{1}{2}\left(\frac{\left(x-p_{x}\right)^{2}}{\sigma_{s,x}^{2}}+\frac{\left(y-p_{y}\right)^{2}}{\sigma_{s,y}^{2}}\right)\right]$$

with parameters $\sigma_{s,x}$ and $\sigma_{s,y}$ specifying the width of the stimulus and $a_{s}$specifying stimulus strength.

The one-dimensional feature attention layer of the model is specified by the following equation:

$$\tau\dot{u}_{fa}\left( y,t \right)= -u_{\text{fa}}\left( y,t \right)+h_{\text{fa}}+\int k_{\text{fa,fa}}\left( y-y^{'} \right)g\left( u_{\text{fa}}\left( y^{'},t \right) \right)dy^{'}+\int dx'\int k_{\text{fa,v}}\left( y-y^{'} \right)g\left( u_{\text{v}}\left( {x^{'},y}^{'},t \right) \right)dy^{'}+\int k_{\text{fa,fc}}\left( y-y^{'} \right)g\left( u_{\text{fc}}\left( y^{'},t \right) \right)dy^{'}+\int k_{\text{fa,fwm}}\left( y-y^{'} \right)g\left( u_{\text{fwm}}\left( y^{'},t \right) \right)dy^{'}+noise$$

where specifies the rate of change of activation across the population of color-selective neurons, *y*, as a function of time, *t*; the constant, τ, sets the time scale; $u_{fa}\left(y,t\right)$ captures the current activation in this layer; and $h_{fa}$ sets the resting level. The feature attention field receives afferent color inputs directly from the two-dimensional visual sensory field, integrated over the spatial dimension, *x*. Activation in *fa* is also influenced by local excitation and broad global inhibition. Activation in the feature attention field is also influenced by feedback from both the feature contrast and feature working memory layers, with the strength and width of the excitatory projections from each field set by an interaction kernel and by spatially correlated noise, as described for one-dimensional projections above.

The feature contrast field is defined by the following equation:

$$\tau\dot{u}_{fc}\left( y,t \right)= -u_{\text{fc}}\left( y,t \right)+h_{\text{fc}}+\int k_{\text{fc,fc}}\left( y-y^{'} \right)g\left( u_{\text{fc}}\left( y^{'},t \right) \right)dy^{'}+\int dx\int k_{\text{fc,v}}\left( y-y^{'} \right)g\left( u_{\text{v}}\left( {x^{'},y}^{'},t \right) \right)dy^{'}+\int k_{\text{fc,fa}}\left( y-y^{'} \right)g\left( u_{\text{fa}}\left( y^{'},t \right) \right)dy^{'}-\int k_{\text{fc,fi}}\left( y-y^{'} \right)g\left( u_{\text{fi}}\left( y^{'},t \right) \right)dy^{'}+noise$$

This equation is very similar to the equation given for the feature attention field, but with slightly different projections. The feature contrast field receives stimulus input from the visual sensory field, integrated along the spatial dimension, and a separate projection from the feature attention field. Additionally, activation is influence by narrow excitatory lateral interactions and inhibition projected from the feature inhibition field. The projection from the feature inhibition field consists of a wide inhibitory component together with a global inhibition term. In each case, the strength and width of projections are set by an interaction kernel convolved with the sigmoid function that determines field output, as above.

The feature inhibition layer is defined by the following equation:

$$\tau\dot{u}_{fi}\left( y,t \right)= -u_{\text{fi}}\left( y,t \right)+h_{\text{fi}}+\int k_{\text{fi,fc}}\left( y-y^{'} \right)g\left( u_{\text{fc}}\left( y^{'},t \right) \right)dy^{'}+\int k_{\text{fi,fwm}}\left( y-y^{'} \right)g\left( u_{\text{fwm}}\left( y^{'},t \right) \right)dy^{'}+noise$$

Activation in the feature inhibition field is driven entirely by inputs from the feature contrast and feature working memory fields. Thus, during the delay period, when no stimulus inputs are present, activation in the feature inhibition field is driven by inputs from peaks in feature working memory. This produces regions of inhibition in the feature contrast field, which underlies the three-layer model’s ability to detect feature changes in the context of change detection. As before, the strength and width of lateral interactions and of projections to the feature inhibition field are determined by an interaction kernel and activation is influenced by spatially correlated noise, as described above.

Finally, the feature working memory field is defined by the following equation:

$$\tau\dot{u}_{fwm}\left( y,t \right)= -u_{\text{fwm}}\left( y,t \right)+h_{\text{fwm}}+\int k_{\text{fwm,fwm}}\left( y-y^{'} \right)g\left( u_{\text{fwm}}\left( y^{'},t \right) \right)dy^{'}+\int dx\int k_{\text{fwm,v}}\left( y-y^{'} \right)g\left( u_{\text{v}}\left( {x^{'},y}^{'},t \right) \right)dy^{'}+\int k_{\text{fwm,fc}}\left( y-y^{'} \right)g\left( u_{\text{fc}}\left( y^{'},t \right) \right)dy^{'}-\int k_{\text{fwm,fi}}\left( y-y^{'} \right)g\left( u_{\text{fi}}\left( y^{'},t \right) \right)dy^{'}+noise$$

Activation in the feature working memory field is driven by excitatory inputs from the visual sensory field and the feature contrast field and is influenced by excitatory lateral interactions and inhibitory input from the feature inhibition field, with the strength and width of each projection set by the interaction kernel.

**Model 1 simulation time course and control inputs**

The time course of each simulation closely matched the structure and timing of the behavioral task performed by participants, with task demands (e.g., the need to generate a response) implemented as external control inputs. Such inputs modulate the behavior of the model through simple changes in field activation levels. We assume such inputs originate from cortical areas involved in cognitive control (such as the prefrontal cortex) or attention at the level of the visual scene, although for simplicity, these areas are not included in the present model (note that a simplified version of attention operating at the level of the scene is implemented in Model 2, described below).

At the beginning of each simulated trial, activation within each field was set to the field resting level. The sample stimulus (or stimuli in the SS3 case) was then activated for 800 ms (in simulation time), and was followed by a 1000 ms blank delay, at which point the test stimulus appeared in the task space. The color wheel was modeled as a sub-threshold, diagonal ridge of activation spanning the width of the spatial and feature dimensions of the visual sensory field. Upon the appearance of the color wheel, activation of a total of 51 units centered on the cued target was boosted by 1 (25 units clock-wise and 25 units counter-clockwise from the cued target). At the same time, the resting level in the feature attention field was incremented from -7 to -4. Together, these events lead to the formation of a single peak of activation in the feature attention field, reflecting the location in hue space of the cued color target. The field output was then projected as a ridge of activation into the visual sensory field, which produced a two-dimensional peak of activation at the *x,y* position in the visual sensory field corresponding to the remembered color and its spatial position in the color wheel. The color wheel remained on for a total of 3000 ms (in simulation time), at which point the color coordinates of the peak in the visual sensory field was read off and the difference from the actual target coordinates was calculated.

**Model 2 architecture and simulation procedures**

The model depicted in Figure 3 and used in Simulation Experiment 2 and the Control Simulation Experiment consisted of the same five DFs featured in Model 1, with the addition of three additional one-dimensional fields—one defined in color coordinates that stands in for attention operating at the level of the scene, and two more that are part of the expanded spatial pathway and are defined in retinal coordinates—and two dynamical nodes that play a role in producing sequential loading of items into WM. In the equations below, the fields shared in common with Model 1 are denoted using the same indices as given above (*v*, *fa*, *fc*, *fi*, and *fwm*), and the new fields and nodes are indicated as follows: *sla* for the new feature attention field that stands in for attention operating at the scene level, *sa* for spatial attention, *ior* for inhibition of return, *pd* for the peak detector node, and *cos* for the Condition of Satisfaction node. As before, parameters of projections between fields are identified by two indices, the first indicating the target, the second the source of the projection.

The scene level attention field is governed by the following equation:

$$\tau\dot{u}_{sla}\left( y,t \right)= -u_{\text{sla}}\left( y,t \right)+h_{\text{sla}}+\int k_{\text{sla,sla}}\left( y-y^{'} \right)g\left( u_{\text{sla}}\left( y^{'},t \right) \right)dy^{'}+\int k_{\text{sla,fa}}\left( y-y^{'} \right)g\left( u_{\text{fa}}\left( y^{'},t \right) \right)dy^{'}+\int k_{\text{sla,fwm}}\left( y-y^{'} \right)g\left( u_{\text{fwm}}\left( y^{'},t \right) \right)dy^{'}+CoS\left( t \right)+noise$$

This field is similar to the feature attention field but its main excitatory inputs come from the feature working memory field, rather than the color-space field. Field activations are also influenced by local excitation and global inhibition, defined by the same interaction kernel, $k\left( y,t \right)$, used for the feature attention field above. Finally, activation in the scene level attention field is inhibited globally by input from the Condition of Satisfaction node, which is activated when a new peak builds in the feature working memory field during stimulus encoding.

The spatial attention field is governed by the following field equation:

$$\tau\dot{u}_{sa}\left( x,t \right)= -u_{\text{sa}}\left( x,t \right)+h_{\text{sa}}+\int k_{\text{sa,sa}}\left( x-x^{'} \right)g\left( u_{\text{sa}}\left( x^{'},t \right) \right)dx^{'}+\int dy\int k_{\text{sa,v}}\left( x-x^{'} \right)g\left( u_{\text{v}}\left( {x^{'},y}^{'},t \right) \right)dx^{'}-\int k_{\text{sa,ior}}\left( x-x^{'} \right)g\left( u_{\text{ior}}\left( x^{'},t \right) \right)dx^{'}+CoS(t)+noise$$

This field is also similar to the feature attention field, with the primary exception that it receives excitatory activation summed along the spatial, rather than feature, dimension of the color-space field. Also, like the scene level attention field, this field receives localized inhibitory inputs from the inhibition of return field and globally inhibitory input from the condition of satisfaction node.

The inhibition of return field is governed by the following equation:

$$\tau\dot{u}_{ior}\left( x,t \right)= -u_{\text{ior}}\left( x,t \right)+h_{\text{ior}}+\int k_{\text{ior,ior}}\left( x-x^{'} \right)g\left( u_{\text{ior}}\left( x^{'},t \right) \right)dx^{'}+\int dy\int k_{\text{ior,v}}\left( x-x^{'} \right)g\left( u_{\text{v}}\left( {x^{'},y}^{'},t \right) \right)dx^{'}+\int k_{\text{ior,sa}}\left( x-x^{'} \right)g\left( u_{\text{sa}}\left( x^{'},t \right) \right)dx^{'}+CoS(t)+noise$$

Like the spatial attention field, this field is defined in retinal coordinates and receives summed excitatory input from the spatial dimension of the color-space field. This field also receives localized input from the spatial attention field and global excitation from the condition of satisfaction node, boosting the resting level of this field and facilitating the building of a peak at the current spatially attended location.

Finally, the two dynamic nodes are governed by the following equations:

$$\tau\dot{CoS}\left( t \right)= -CoS\left( t \right)+h_{\text{CoS}}+c_{CoS,CoS}g\left( CoS\left( t \right) \right)+c_{CoS,PD}g\left( PD\left( t \right) \right)+noise$$

$$\tau\dot{PD}\left( t \right)= -PD\left( t \right)+h_{\text{PD}}+c_{PD,PD}g\left( PD\left( t \right) \right)+\int c_{PD,sla}g\left( u_{\text{sla}}\left( y^{'},t \right) \right)dy^{'}+noise$$

Each node has a resting level and self-excitation. In addition, the *PD* node is driven by activation in the scene-level attention field, while the *CoS* node receives input from the *PD* node.

**Model 2 simulation time course and control inputs**

As with Model 1, the time course of each simulation matched the structure and timing of the behavioral task performed by participants, and task demands were implemented as external control inputs, as described above. The sequence of events in a trial was modeled in an identical manner as described for Model 1 above, up until the point that the color wheel appeared and a response was generated. As before, the color wheel was modeled as a sub-threshold, diagonal ridge of activation spanning the width of the spatial and feature dimensions of the visual sensory field. Upon the appearance of the color wheel, activation of a total of 51 units centered on the cued feature value in the scene level attention field was boosted by 1 (25 units clock-wise and 25 units counter-clockwise from the cued target). However, instead of providing this boost directly to the feature working memory field, the boost was applied to the scene level attention field (Note: this boost is visible in the bottom-most panel of Figure 3). Excitatory feedback from the scene level attention field to feature working memory then increased the amplitude of the peak at that location. As in Model 1, at the same time, the resting level in the feature attention field was incremented from -7 to -4 (that is, the resting level of the field was shifted upwards). Together, these events lead to the formation of a single peak of activation in the feature attention field, reflecting the location in hue space of the recalled color target. The field output was then projected as a ridge of activation into the visual sensory field, as before, which produced a two-dimensional peak of activation at the *x,y* position in the visual sensory field corresponding to the remembered color and its spatial position in the color wheel. Emerging activation in CS boosted activation at the spatial location of the recalled color target in the SA field, implementing a form of biased competition. The color wheel remained on for a total of 3000 ms (in simulation time), at which point the color coordinates where the model ‘clicked’ on the color wheel (i.e., the color presented at the spatial location of the peak in the spatial attention field) was read off and the difference from the actual target coordinates was calculated.

**Model parameter tuning procedures**

The models described here build on and incorporate elements of a few different previous models. The core three-layer model was first developed and the parameters tuned as part of a PhD thesis (submitted May 2008) by the first author (see Johnson, Spencer , & Schöner, 2009; Johnson, Spencer, Luck, & Schöner, 2009). This model was incorporated in Model 1, described here with the addition of a feature attention field and a 2D color-space field to support recall responses. For these simulations, 200-300 simulations were run per model iteration, and RMSE was calculated relative to the empirical data, with the goal of lowering RMSE across iterations. After each iteration, we documented problems with the current results, formed hypotheses about how to improve fit (e.g., which params matter), and ran additional simulations to explore the impact of changes. This process was then repeated over a series simulation batches. If the chosen modifications lowered RMSE, we iterated around that region of parameter space in an attempt to improve fit across all conditions and parameters. If, after several batches of simulations, the results were not promising, we reverted back to the best previous parameter set and tried out the next hypothesis. The simulator was updated and an interface with the mixture modelling approach was implemented in 2013. The results presented here are very similar to that model simulator, which was migrated to the COSIVINA framework and modified to incorporate the MemToolbox in 2014.

Given a few years to stew on the result sof Model 1 and some of its shortcomings, in 2020, we developed the Model 2 simulator, which added several additional fields, and a couple of individual nodes, with the goal of implementing sequential consolidation in WM. These elements had been incorporated in at least one previous model within this framework, and initial parameters were taken directly from these simulators. Insertion into Model 1 required an initial tuning of several parameters, just to to stabilize the model’s behavior with the new additions (see italicized parameters in Tables 1-3). Specific modifications included increasing the strength of global inhibition in the feature attention field and stronger interactions between both FWM and feature attention, and between the feature attention and feature contrast fields. These connections support sequential consolidation and recall when activation in FWM is boosted around the cued item at test. Additionally, we also needed a strong consolidation signal (FWM 🡪scene level attention) and strong recall boost (scene level attention🡪FWM). Stabilizing the model’s behavior required a total of 48 batches of simulations of Model 2.

Once Model 2 behavior was stabilized, we created a new Model 1 simulator to make sure the GUIs and model details were the same as Model 2. We also fixed a few inconsistencies across simulators and re-simulated Model 1 and Model 2 such that Model 1 replicated previous findings and Model 2 was in the right ballpark. We then iterated the parameters of Model 2 (see bold parameters in Tables 1-3) around the parameter values settled on in the last step, described above. The key issue we were trying to address was low overall s.d., and the mismatch between the guessing rate observed for the Unique target observed in Simulation Experiment 2, which was much lower than what was observed in Experiment 1. To increase s.d., we increased the amplitude of the spatially correlated noise added to FWM, the visual sensory field, and the feature attention field. This achieved the desired effect of increasing overall recall variability, but resulted in the formation of spurious peaks in FWM on many simulated trials. To counteract this, we reduced the noise level in the FWM field.

Next, we explored the connections between the feature attention field and the visual sensory field, which should have an influence on the precision of recalled responses mapping to color. Stronger input from feature attention to the visual attention field increased the difference in s.d. between the Unique and Close color conditions. Additionally, broader input from feature attention to the visual sensory field and vice versa increased s.d. globally, compared to Model 1.

These adaptations were finalized over seven iterations. We then ran an additional 30 simulations in an attempt to improve model fit by incrementing the values of several parameters around their current values, with no improvement over Model 2 Version 7. Finally, we took Model 2-v.7 and compared it to Model 1-v. 7 and we equalized a few final parameters without impacting Model 1 performance. The final version is named Model 1-v9 and is available via the following URL: https://github.com/cosivina/dft_inprogress/tree/master/Johnson_VWM-Repulsion.

**Calculation of number of free parameters for each model**

The main text reports the Akaike Information Criterion (AIC) for each model. This model gives an estimate of how well each model fits the data across conditions, while accounting for overall model complexity, which is defined as the number of parameters that are free to vary (denoted by *k* in the equation). For Model 1, the total number of parameters that were free to vary came out to 49. As shown in Supplementary Tables 1 and 2, there were five (5) parameters specifying the resting level (*h*) of each field, 12 parameters specifying the strengths (c_exc_ and c_inh_) and widths (σ_exc_ and σ_inh_) of interactions within each field, 26 parameters that control the strength and width of connections between fields, and six (6) additional parameters shown in Supplementary Table 3. Note that the time constants (τ) and sigmoidal threshold values (ß) were not modified, and thus were not included in the tally of free parameters for Model 1.

For Model 2, we calculated a total of 56 parameters that were free to vary. Many of the parameters in Model 2 were set to default values based on Model 1 simulations, or, for the new model components, to default values used in previous studies. Specifically, the resting level (*h*) of each field was carried over from Model 1, the width of excitatory interactions within each field was held constant, and we used a default level of global inhibition established in previous work for the added scene level and spatial attention fields. The width of inhibitory interactions in the IOR field was also fixed based on previous research. Additionally, the sigmoidal threshold values (ß) were carried over from Model 1, and the time constants (τ) were scaled to speed up the simulations, but were not adjusted otherwise. Thus, all of these parameters were not included in the tally of free parameters, because they were not modified during parameter tuning. Of the remaining parameters, a total of seven (7) were modified during the iterative parameter tuning process for Model 2 described above. These parameters are printed in bold font in Supplementary Tables 2-3, and include the strength (c_exc_) and width (σ_exc_) of excitatory connections from the visual sensory field (v) to the feature attention (fa) field, and from fa back to v, the noise strength in both fa and fwm, and, finally, the strength of the recall input boost from the scene level attention field to fwm.

**Supplementary Results**

As noted in the main text, the finding of increased s.d. for the Close versus Unique target colors could have arisen as a result of an increased tendency on the part of participants to report the uncued close color, instead of the close target, at test. To determine whether such “swap” errors occurred at a greater rate in the Unique versus CW and CCW close color conditions, we conducted another mixture model analysis using the three component “swap” model (with bias) proposed by Bays and colleagues (Bays et al., 2009; Bays & Husain, 2008), as implemented in the MemToolbox and closely inspected the aggregate response distributions for each condition. We also took a look at the relationship between the guess rate (g) and standard deviation (sd) parameter estimates for each condition to determine whether the elevated s.d. in the CW and CCW conditions and the increased guessing in the Unique condition could reflect a trade-off between these parameters, rather than a true effect of metric similarity. Note that the model fits shown here were based on data for each condition aggregated across participants, rather than fit to individual participant data and then averaged together, as in the analysis reported in the main text.

Results of the mixture model analysis using the swap model (w/bias) are depicted in Figure S1, which shows the experimental data and model fits (right side of each plot) together with the maximum a posteriori and credible intervals of the model values inferred from the data (left side of each plot). First, it should be noted that the values given for μ (mean bias) and the overall pattern of guessing (g) and s.d. across conditions was very similar to the values reported for Experiment 1 in Figure 2, using the standard mixture and individual participant-level data. This analysis also revealed a much higher likelihood of non-target responses in both of the close color conditions (proportion NT responses = .06), compared to the unique condition (pNT = .005). Taking a look at the response distributions reveals clear clustering of responses around the Unique non-target item in each condition (-170 degrees for the CCW condition, and 170 degrees for the CW condition). However, there is no obvious clustering of responses around the close non-target item. Instead, responses were more likely to fall in the first 4 or 5 bins moving away from, rather than towards, the other close item (i.e., spanning 0 to ~+50 degrees or so for the CW condition, and 0 to -50 for the CCW condition). Additionally, comparison of the response histograms for the Unique item and the two Close items reveals that a larger proportion of responses fell within the four bins spanning the interval -20 to +20 degrees around the actual target value (i.e., in the four bins surrounding 0-error) in the Unique condition (59.79% percent of responses) compared to either the CW (54.53%) or CCW (55.04%) targets. Overall, these patterns are consistent with the conclusion that responses are shifted away from the nearby close color in the CW and CCW conditions, and are more likely to cluster near the actual target value in the Unique condition than in either of the two Close condition


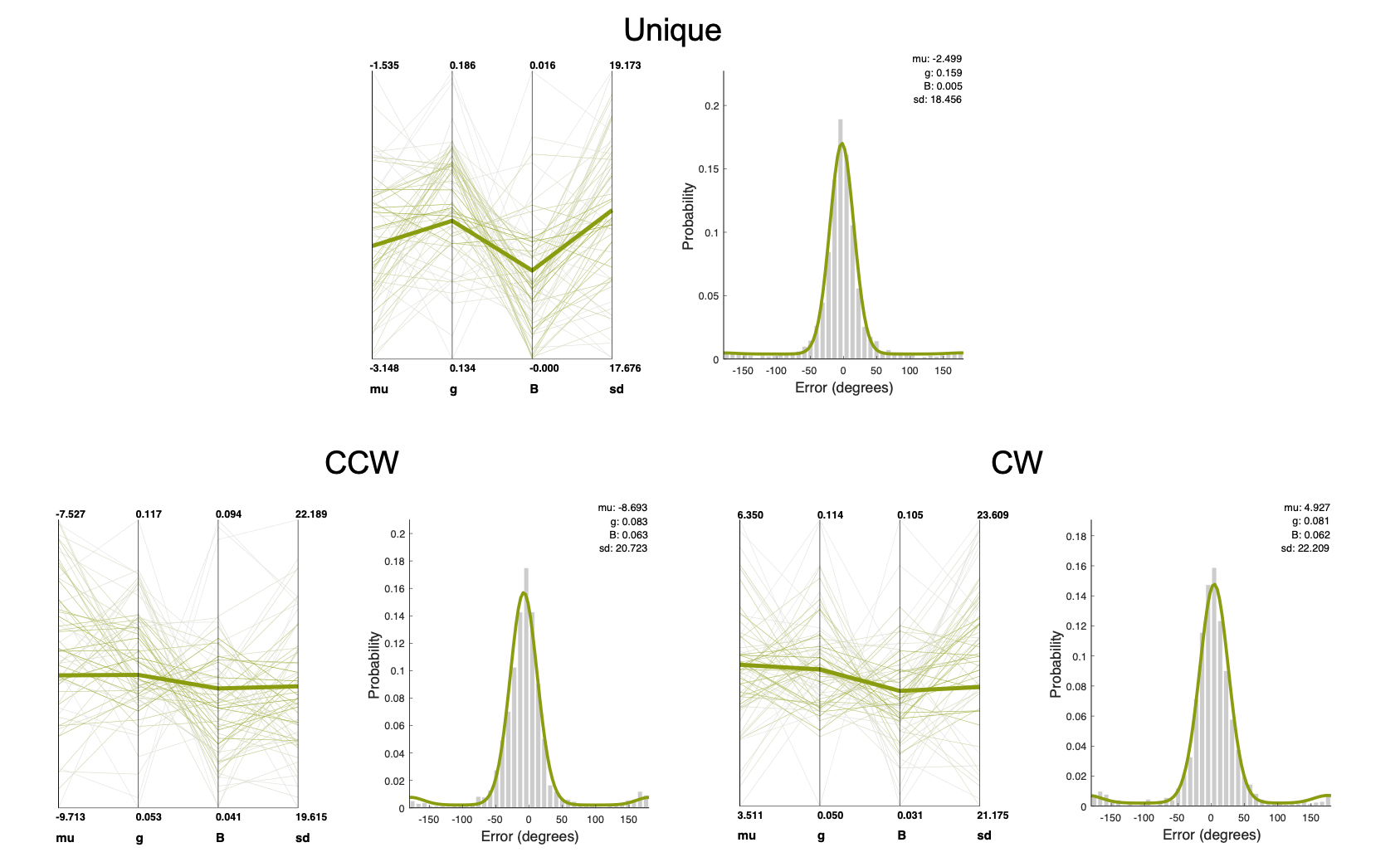


Supplementary Figure 1. Probability distributions of aggregated participant responses (light gray bars) and model fits (right side of each panel) together with the maximum posteriori and credible intervals (left side) of parameter values generated by MemToolbox for each condition in Experiment 1.

To assess whether observed differences in s.d. and guess rate (g) across conditions reflects a trade-off between these parameters, we took a look at the plots of correlations between parameters that MemToolbox generates (see Figure S2). Once again, this is for model fits obtained using data aggregated across participants. The off-diagonal shows the full posterior distribution of parameter estimates generated by the model for g and s.d., and the diagonal shows the heatmap for each pair of parameters. Looking at these figures, there appears to be a negative correlation between g and s.d., which suggests that the data in each condition are equally consistent with a slightly higher g and a slightly lower s.d., or with a slightly lower g and higher s.d. Note, however, that the credible ranges given for these parameters for the Unique versus CW/CCW conditions are entirely non-overlapping (the ranges for each condition can also be seen in the left side of each panel in Figure S1). For the Unique condition, credible model estimates for g ranged from .134-.186 (Mean = .159), versus .053-.117 (M = .083) and .05-.114 (M = .081) in the CCW and CW condition, respectively. Similarly, s.d. ranged from 17.67-19.17 (M = 18.45) in the Unique condition, versus 19.615-22.189 (M = 20.72) and 21.175-23.609 (M = 22.21) in the CCW and CW condition, respectively. Thus, although there does seem to be a trade-off between g and s.d., and the true value for each of these parameters could be somewhat higher or lower across conditions, small variations in one or the other direction likely would not eliminate the overall difference in s.d. between the Unique and the Close color conditions.


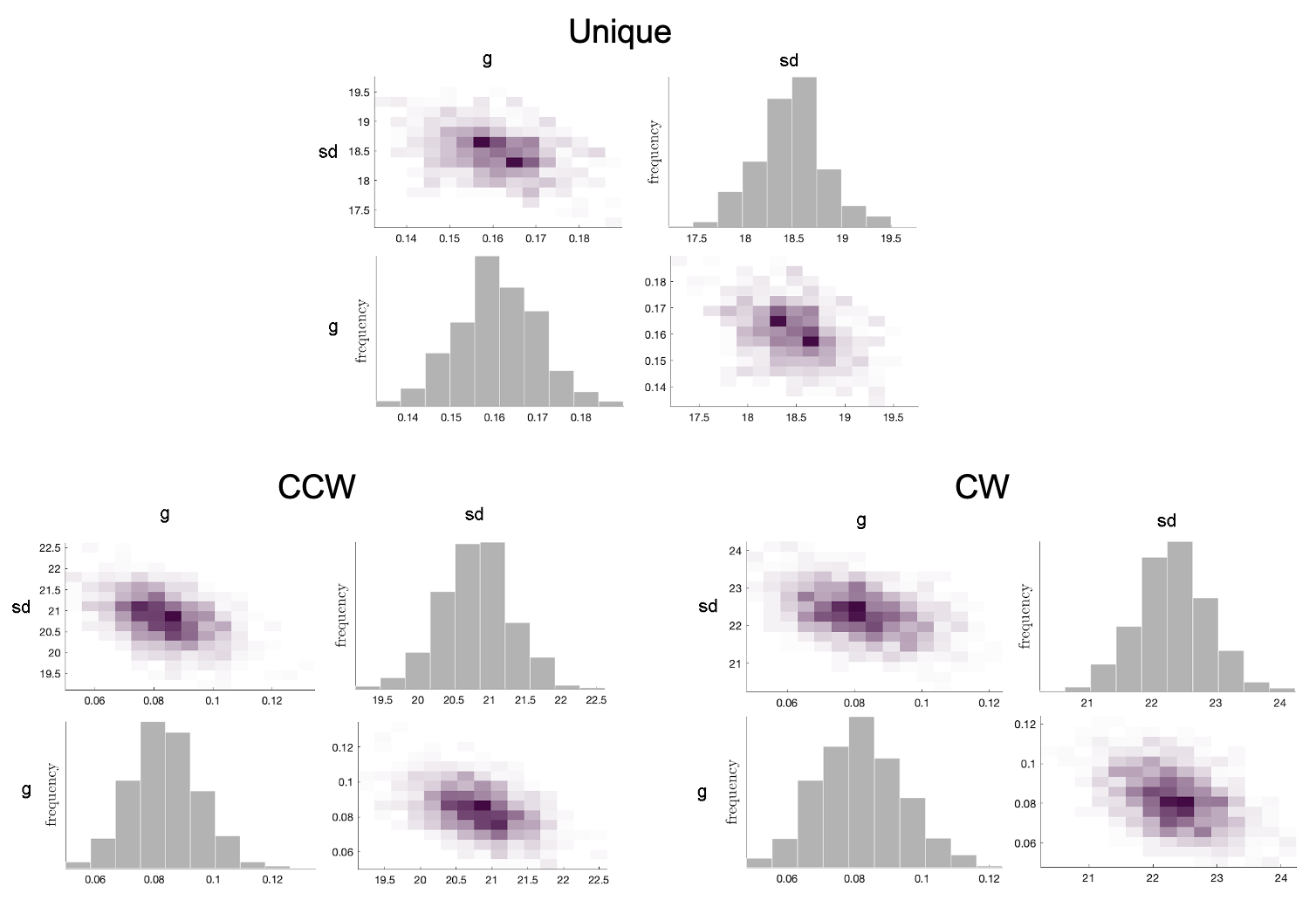


Supplementary Figure 2. Heatmaps depicting the relationship between parameter estimates for g (guessing rate) and s.d. across conditions of Experiment 1.
