# Supplementary material for: Neural interactions in working memory explain decreased recall precision and similarity-based feature repulsion
Source: Sci Rep. 2022 Oct 22;12:17756. doi: 10.1038/s41598-022-22328-4 (PMC9588047; doi:10.1038/s41598-022-22328-4)
Supplement: Supplementary file 4 — Supplementary Table 1. [file 41598_2022_22328_MOESM4_ESM.docx]

Supplementary Table 1

Field parameters and parameters of lateral interactions

| Field index | τ | *h* | ß | c_exc_ | σ_exc_ | c_inh_ | σ_inh_ | c_gi_ |
| --- | --- | --- | --- | --- | --- | --- | --- | --- |
| **Model 1** |  |  |  |  |  |  |  |  |
| v(ftr,spt) | 40 | -5 | 5 | 7.2/7.2 | 4/4 | -7.5 | 8/8 | -.005 |
| fa | 40 | -7 | 5 | 4 | 4 | - | - | -0.5 |
| fc | 40 | -7 | 5 | 2 | 3 | - | - | - |
| fi | 5 | -12 | 5 | - | - | - | - | - |
| fwm | 40 | -4 | 5 | 3.15 | 3 | - | - | - |
| **Model 2** |  |  |  |  |  |  |  |  |
| v(ftr,spt) | 12 | -5 | 5 | 7.2/7.2 | 4/4 | -7.5 | 8/8 | -.0175 |
| fa | 12 | -7 | 5 | *9* | 4 | - | - | *-1.0* |
| fc | 12 | -7 | 5 | 2 | 3 | - | - | - |
| fi | 3 | -12 | 5 | - | - | - | - | - |
| fwm | 12 | -4 | 5 | *3.7* | 3 | - | - | - |
| sla | 12 | -5 | 5 | 4 | 4 | - | - | -0.5 |
| sa | 12 | -5 | 5 | 12 | 4 | - | - | -1.0 |
| ior | 12 | -5 | 5 | 20 | 4 | -15 | 8 | - |
