# Supplementary material for: Neural interactions in working memory explain decreased recall precision and similarity-based feature repulsion
Source: Sci Rep. 2022 Oct 22;12:17756. doi: 10.1038/s41598-022-22328-4 (PMC9588047; doi:10.1038/s41598-022-22328-4)
Supplement: Supplementary file 5 — Supplementary Table 2. [file 41598_2022_22328_MOESM5_ESM.docx]

Supplementary Table 2

Parameters of interactions between fields

| Projection index | c_exc_ | σ_exc_ | c_inh_ | σ_inh_ | c_gi_ |
| --- | --- | --- | --- | --- | --- |
| **Model 1** |  |  |  |  |  |
| fa,v | 1 | 4 | - | - | - |
| fc,v | 3 | 1 | - | - | - |
| fwm,v | 0.2 | 1 | - | - | - |
| v,fa | 2.5 | 4 | - | - | - |
| fc,fa | 1 | 5 | - | - | - |
| fa,fc | 1 | 5 | - | - | - |
| fi,fc | 2 | 10 | - | - | - |
| fwm,fc | 1.5 | 5 | - | - | - |
| fc,fi | - | - | -1.15 | 24 | -0.05 |
| fwm,fi | - | - | -0.325 | 28 | -0.02 |
| fa,fwm | 5 | 4 | - | - | - |
| fi,fwm | 1.95 | 13 | - | - | - |
| **Model 2** |  |  |  |  |  |
| fa,v | **1.5** | **20** | - | - | - |
| fc,v | 1.5 | 4 | - | - | - |
| fwm,v | 0.1 | 4 | - | - | - |
| sa,v | 0.42 | 4 | - | - | - |
| ior,v | 0.15 | 4 | - | - | - |
| v,fa | **8** | **30** | - | - | - |
| fc,fa | *7.5* | 4 | - | - | - |
| fwm,fa | *14* | 4 | - | - | - |
| sla,fa | *9* | 4 | - | - | - |
| fa,fc | *6* | 4 | - | - | - |
| fi,fc | 1 | 10 | - | - | - |
| fwm,fc | 1.5 | 5 | - | - | - |
| fc,fi | - | - | -0.85 | 15 | -0.05 |
| fwm,fi | - | - | -0.325 | 28 | -0.02 |
| fa,fwm | *8.5* | 12 | - | - | - |
| fi,fwm | 1.85 | 13 | - | - | - |
| sla,fwm | *6* | 5 | - | - | - |
| fwm,sla | *11* | 5 | - | - | - |
| pd,sla | 3 | - | - | - | - |
| ior,sa | 2 | 4 | - | - | - |
| v,sa | 2 | 4 | - | - | - |
| sa,ior | -10 | 4 | - | - | - |
| ior,cos | 2.25 | - | - | - | - |
| fa,cos | -10 | - | - | - | - |
| sla,cos | -10 | - | - | - | - |
