# Supplementary material for: Neural interactions in working memory explain decreased recall precision and similarity-based feature repulsion
Source: Sci Rep. 2022 Oct 22;12:17756. doi: 10.1038/s41598-022-22328-4 (PMC9588047; doi:10.1038/s41598-022-22328-4)
Supplement: Supplementary file 6 — Supplementary Table 3. [file 41598_2022_22328_MOESM6_ESM.docx]

Supplementary Table 3

Additional parameters modulated to improve fit of model 1 or 2

| Description | Value |
| --- | --- |
| **Model 1** |  |
| Noise strength fa | 1.2 |
| Noise strength fwm | 4 |
| Strength of recall input boost | 4.5 |
| Color wheel input strength | 4.5 |
| Visual boost | 1.5 |
| Attention boost | 3 |
| **Model 2** |  |
| Noise strength fa | **1.6** |
| Noise strength fwm | **3.5** |
| Strength of recall input boost | **13** |
| Color wheel input strength | 4 |
| Visual boost | 4 |
| Attention boost | 2.9 |
